# Impact of wheat aleurone on biomarkers of cardiovascular disease, gut microbiota and metabolites in adults with high body mass index: a double-blind, placebo-controlled, randomized clinical trial

**DOI:** 10.1007/s00394-022-02836-9

**Published:** 2022-03-05

**Authors:** Francesca Fava, Maria M. Ulaszewska, Matthias Scholz, Jan Stanstrup, Lorenzo Nissen, Fulvio Mattivi, Joan Vermeiren, Douwina Bosscher, Carlo Pedrolli, Kieran M. Tuohy

**Affiliations:** 1 Department of Food Quality and Nutrition, Research and Innovation Centre, Fondazione Edmund Mach (FEM), San Michele all’Adige, Trento, Italy; 2 I.R.C.C.S. Ospedale San Raffaele, Milan, Italy; 3 DISTAL, Alma Mater Studiorum, Università di Bologna, UOS Cesena, P.zza Goidanich 60, 47521 Cesena, FC Italy; 4 Dipartimento di Biologia Cellulare, Computazionale e Integrata, CIBIO, Trento, Italy; 5 Cargill R&D Centre Europe, Vilvoorde, Belgium; 6 U.O.S. di Dietetica E Nutrizione Clinica, Ospedale S. Chiara, APSS, Trento, Italy; 7 Department of Nutrition, Exercise and Sports, Copenhagen University, Copenhagen, Denmark; 8 School of Food Science and Nutrition, University of Leeds, Leeds, LS2 9JT UK

**Keywords:** Aleurone, Homocysteine, Gut microbiota, Biomarkers of intake

## Abstract

**Purpose:**

Aleurone is a cereal bran fraction containing a variety of beneficial nutrients including polyphenols, fibers, minerals and vitamins. Animal and human studies support the beneficial role of aleurone consumption in reducing cardiovascular disease (CVD) risk. Gut microbiota fiber fermentation, polyphenol metabolism and betaine/choline metabolism may in part contribute to the physiological effects of aleurone. As primary objective, this study evaluated whether wheat aleurone supplemented foods could modify plasma homocysteine. Secondary objectives included changes in CVD biomarkers, fecal microbiota composition and plasma/urine metabolite profiles.

**Methods:**

A parallel double-blind, placebo-controlled and randomized trial was carried out in two groups of obese/overweight subjects, matched for age, BMI and gender, consuming foods supplemented with either aleurone (27 g/day) (AL, *n* = 34) or cellulose (placebo treatment, PL, *n* = 33) for 4 weeks.

**Results:**

No significant changes in plasma homocysteine or other clinical markers were observed with either treatment. Dietary fiber intake increased after AL and PL, animal protein intake increased after PL treatment. We observed a significant increase in fecal *Bifidobacterium* spp with AL and *Lactobacillus* spp with both AL and PL, but overall fecal microbiota community structure changed little according to 16S rRNA metataxonomics. Metabolomics implicated microbial metabolism of aleurone polyphenols and revealed distinctive biomarkers of AL treatment, including alkylresorcinol, cinnamic, benzoic and ferulic acids, folic acid, fatty acids, benzoxazinoid and roasted aroma related metabolites. Correlation analysis highlighted bacterial genera potentially linked to urinary compounds derived from aleurone metabolism and clinical parameters.

**Conclusions:**

Aleurone has potential to modulate the gut microbial metabolic output and increase fecal bifidobacterial abundance. However, in this study, aleurone did not impact on plasma homocysteine or other CVD biomarkers.

**Trial Registration:**

The study was registered at ClinicalTrials.gov (NCT02067026) on the 17th February 2014.

**Supplementary Information:**

The online version contains supplementary material available at 10.1007/s00394-022-02836-9.

## Introduction

Cross-sectional studies have shown that whole grain cereal consumption can reduce the risk of cardiovascular disease (CVD) [1] and reduce systemic inflammation, linked to many chronic diet associated diseases [2]. Wholegrain cereal intake has also been shown to modulate gut microbiota composition and activity. Conversely, microbial metabolism of whole grain bioactives such as fiber and polyphenols, modulates their bioavailability and biological activity [3–6]. However, we still do not fully understand the relative contributions of plant-derived bioactives or microbiome-related activities to the observed health effects of whole grain cereals.

Aleurone is a wheat grain fraction composed of a single cell layer that constitutes the outermost portion of the endosperm and contains many of the beneficial substances including ferulic acid and other phenolic acids, fibers (mainly arabinoxylans, β-glucans, and cellulose), minerals, betaine and vitamins (e.g. folate). Dietary supplementation with aleurone (10% of total food intake) has been shown to reduce symptoms of hypertension and hyperglycaemia in spontaneously hypertensive rats [7]. Aleurone consumption (10%) in high fat fed obese mice also significantly lowered pro-inflammatory cytokines, while modulating the gut microbiota, increasing the abundance of *Bifidobacterium* and *Lactobacillus* species [8].

Aleurone is rich in bioavailable folate and betaine, and both short-term and long-term clinical studies have shown that aleurone-enriched foods can significantly increase plasma methyl donors and lower plasma homocysteine, a recognized marker of CVD [9–11]. In a parallel, single-blinded intervention study, 39 healthy, overweight individuals (BMI ≥ 25 kg/m^2^, 45–65 years old) consuming 27 g/day of aleurone cereal (“Aleurone Standard Preparation 2”) showed significantly elevated plasma concentrations of betaine, betaine metabolites (dimethylglycine, and methionine) and lowered plasma homocysteine levels compared to a control diet [11]. Aleurone consumption also significantly lowered LDL cholesterol and C reactive protein (CRP) [11, 12]. No changes were observed in other inflammation markers, plasma antioxidants status, or endothelial function and the impact on the gut microbiota was not reported. Similarly, small phenolic acids in blood or urine which derive from host:microbiota co-metabolic processing of aleurone polyphenols were not measured.

Aleurone is also rich in fibers such as arabinoxylans, which may support microbial carbohydrate fermentation, modulating microbiota community composition and increasing production of short chain fatty acids (SCFA) [13]. Previous in vitro studies using the Simulator of the Human Intestinal Microbial Ecosystem (SHIME) showed that fermentation of aleurone arabinoxylans by the human gut microbiota significantly increased numbers of bifidobacteria, decreased staphylococci and upregulated SCFA production [14]. We previously demonstrated that aleurone is fermented in vitro by the human gut microbiota and induces an increase in bifidobacteria, *Dorea* and butyrate-producing *Roseburia* spp and decreases the relative abundance of bacteria commonly considered detrimental for human health, specifically *Bilophila*, *Escherichia* and Parabacteroides [15].

Prebiotics are defined as substrates that are selectively utilized by host microorganisms conferring a health benefit [16]. However, whether aleurone can be considered a prebiotic remains to be demonstrated in humans, and specifically, it is unclear whether aleurone intake can increase fecal bifidobacterial abundance as shown for arabinoxylans and arabinoxylooligosaccharides [14, 15]. Microbial metabolism of aleurone may also increase bioavailability of ferulic acid and other phytonutrients bound to indigestible carbohydrates in the food matrix, while generating small phenolic bioactive compounds and SCFA. However, few human studies have explored the ability of aleurone to modify both fecal microbiota composition and metabolic output in terms of SCFA and also small phenolic acids. This randomized, double-blind and placebo-controlled study aimed to evaluate whether wheat aleurone-rich food supplementation lowers plasma homocysteine in subjects at increased metabolic risk (i.e. overweight). This was the primary objective. Secondary to this, common clinical parameters related to CVD risk were also measured. The study further explored the impact of aleurone supplementation on the gut microbiota. We accurately quantified fecal bifidobacteria and lactobacilli, which are commonly stimulated by prebiotics, using quantitative PCR and Fluorescent in situ hybridization methodologies. We also measured the impact of aleurone on whole fecal microbiota community structure using 16S rRNA based metataxonomics and on metabolite profiles which may be related to microbiota activities using untargeted and targeted metabolomics approaches. Fasting blood samples were chosen to assess changes in CVD-related clinical parameters, in line with standard medical practice. Microbial metabolites on the other hand, were measured in both fasting plasma and in 24 h urine collections to gain insight into host:microbiome co-metabolic processing of aleurone-derived metabolites irrespective of metabolite clearance from blood. The study was conducted jointly by researchers at Fondazione Edmund Mach (San Michele all’Adige, Italy), and the Unità Ospedaliera Sanitaria di Dietetica e Nutrizione Clinica of the Ospedale S. Chiara, Azienda Provinciale per i Servizi Sanitari della Provincia Autonoma di Trento (Italy) and the study was supported by Cargill R&D Center Europe (Vilvoorde, Belgium).

## Subjects and methods

### Ethical approval

The study was authorized by the Comitato Etico per le Sperimentazioni Cliniche, APSS, Trento (ID 52,883,383, 10/10/2014, Class. II.9) and is registered at clinicaltrials.gov (NCT02067026). The study was conducted in line with the Declaration of Helsinki of 1975 as revised in 1983.

### Study design

The study was set up as a placebo-controlled, randomized, double-blind parallel trial with 2 test foods, wheat aleurone-rich foods or placebo foods (cellulose).

Participants were recruited primarily from the local (Trento, Italy) community upon spontaneous response to public advertisements and publicity materials. Initial phone contacts and interviews, subsequent appointments for medical examinations and blood collections, were performed by the principle investigator (Francesca Fava). Recruitment interviews were conducted according to a standardized pre-agreed protocol under the medical supervision of Professor Carlo Pedrolli, Head of Nutrition and Dietetics, Santa Chiara Hospital, Trento. Medical examinations were conducted by Prof Pedrolli and blood collection occurred at the outpatient blood sampling point at Santa Chiara Hospital. Participants were informed about the study aims and procedures and were pre-screened on the basis of the following inclusion and exclusion criteria: inclusion criteria: aged 18–65 years; BMI > 27 kg/m^2^; exclusion criteria: fasting blood glucose > 300 mg/dl, triglycerides > 500 mg/dl, uncontrolled hypertension (blood pressure [BP] > 160/100 mm Hg under antihypertensive therapy [17]), cardiovascular disease (myocardial infarction, percutaneous transluminal coronary angioplasty or coronary artery bypass grafting, unstable angina pectoris, stroke, peripheral arterial disease), hypo- or hyperthyroidism, acute inflammatory diseases, severe gastrointestinal diseases, heart, liver, renal or pulmonary failure or other life threatening disease with prognosis < 5 years, chronic use of systemic corticosteroids, anticoagulants, anti-inflammatories, or lipid lowering and antidiabetics drugs, treatment within the previous 6 weeks with any medication that is known to affect lipoprotein levels or fecal microbiota (specifically, antibiotics), food intolerances, alcohol intake > 5 drinks per day or use of narcotic substances, use of antioxidant vitamin or mineral supplements, special diet, pregnancy, tobacco smoking (occasional smoking was tolerated). If eligible, they signed the informed consent and entered the study with visit V0, performed at the clinic between 8.00 and 9.00 a.m., where a clinical and biochemical evaluation of the health status were performed. If subjects were eligible for the study, at visit V1 (2 weeks after V0, run-in period) they were included in the trial and randomized to receive the active supplementation (aleurone 27 g per day) or placebo for 4 consecutive weeks, ending with V2. Participants were advised to avoid changes in their habitual diet, and not to consume prebiotics, probiotics or food supplements of any kind.

Clinical examinations, tests, and blood drawing were performed after an overnight fasting at the start of the run-in phase (V0), at inclusion visit (V1) and at the end of supplementation period (V2). Stool and twenty-four hours (24 h) urine collection were performed, at visit V1 and V2. A 3-day food diary questionnaire was filled in by volunteers before visit V1 and V2.

### Supplementation

Food products (bread, breakfast biscuits, ready to eat cereals) were manufactured by Bühler AG (Switzerland), Sonneveld Group BV (The Netherlands) and by a local bakery (Panificio Brugnara, Pergine Valsugana, Trento, Italy), using aleurone produced by Cargill (Horizon Milling, USA), now Ardent Mills (USA), under license of Bühler. The test products/foods were identical in terms of structure, texture, and color and had very similar organoleptic characteristics. Foods were packaged in identical material marked with expiry date and study number/code. Cargill staff monitored the safe production of the test foods, but were blinded as to treatment allocation and randomization of subjects.

At V1 participants were randomized to receive supplementation with either wheat aleurone-rich food (AL) or placebo foods (PL) for four weeks in a double-blind manner. Randomization was conducted according to the randomization criteria of BMI, age and gender. Participants were allocated to one of the experimental groups by a member of staff not involved in other study procedures, to create two study groups that did not differ significantly for the specified randomization criteria, using student’s *t* test. At randomization visit, a sequential 5 digit number was assigned to each volunteer and used to label study samples and documents. Study foods consisted in one portion of bread (2 × 58 g buns containing 18 g aleurone/portion), one portion of biscuits (2 × 15 g biscuits containing 9 g aleurone/portion) and one portion of breakfast cereals (36 g of ready to eat—RTE—cereals, containing 9 g aleurone/portion). Placebo foods contained the same ingredients as the active foods except with cellulose substituting for aleurone and were developed to be similar in calorie content, size, shape and color. Study products, both treatment and placebo foods, were packaged in opaque envelopes that were labeled by the manufacturers with random letters, corresponding to either Aleurone or placebo. Unblinding of the study was performed after study completion and data analysis. Volunteers were told to consume one portion of bread and in addition, they were given the choice to consume either biscuits or cereals, although they were asked to consume each combination (i.e. bread plus biscuits and bread plus cereals) for an equal number of times during the supplementation period. Study products were consumed in different meals of the day. The total daily intake of aleurone was 27 g/day. Study products were provided in product packages that were labeled in the local language with the study code number, batch number, instructions for use, allergen statement, storage instructions and expire dates. The composition of study foods is summarized in Table 1.

**Table 1:** Composition of study foods

	Bread (116 g)		Biscuits (30 g)		Cereals (36 g)	
Treatment	AL	PL	AL	PL	AL	PL
Calories (kcal)	234.3	216.7	183.6	178.8	118.44	118.08
Proteins (g)	11.99	12.1	3.12	3.68	4.968	5.004
carbohydrates (g)	42.24	38.50	14.72	13.72	22.50	22.28
Fats (g)	1.98	1.65	12.52	12.16	0.94	1.01
Dietary fiber (g)	10.67	12.98	4.84	5.96	4.68	3.744
Aleurone extract (g)	18	–	9	–	9	–

*AL* aleurone-enriched foods, *PL* Placebo, –  none

### Clinical evaluation

A 12-lead electrocardiogram (ECG) was performed at screening. Electrocardiographs were reviewed and interpreted by Dr Carlo Pedrolli, U.O.S. di Dietetica e Nutrizione Clinica, St Chiara, APSS Trento. Arterial blood pressure (BP) was measured according to the guide-lines for hypertension of ISH/WHO 2004. The ‘OMRON HEM705CP Oscillometric blood pressure monitor’ was used. Subjects were kept resting and seated for 10 min before measuring blood pressure on the right arm and with the arm angle at the same level of the heart. Weight and height were measured on a standard scale, with an attached altimeter for height measurements and were recorded as equal to the closest 100 g and 1 cm, respectively. Measures were carried out on subjects without shoes, coats or heavy dressing. BMI was calculated as kg/m^2^. Waist (umbilical) circumference was measured according to National Institutes of Health, National Heart, Lung, and Blood Institute [18]. In practice, with the subject standing erect with the abdomen relaxed, the arm at the sides and the feet together, the waist circumference was measured, at the nearest 0.1 cm, at a level midway between the lower rib margin and iliac crest with the tape all around the body in horizontal position. Hip circumference was measured at bitrochanteric level.

### Questionnaires

All the information was collected and recorded on the hospital database, including personal identification, medical history, and information on risk factors for CVD, drug use, physical activity, and dietary habits. 3-day food diaries (i.e. food intake records for the three consecutive days preceding the study visits) were analyzed using the software Terapia Alimentare Dietosystem (DS medica S.r.l) and the Italian databases of food composition (LARN) [19]. Daily stool frequency, stool consistency and intestinal habit (abdominal pain, flatulence and discomfort) records were collected from participants [20].

### Sample collection

Blood (≤ 30 ml) was collected from an antecubital vein, with minimal stasis between 8.00 and 9.00 a.m. after 12 h fasting, from subjects who refrained to smoking for at least 6 h. 24 h urine collections w from each participant were measured and 4 aliquots of 5 ml were stored at − 80 °C. Feacal samples were processed within 2 h of defecation. Briefly, 1 g aliquotes of feces was stored at − 80 °C for later DNA extraction. Fecal samples were also processed for further fluorescent in situ hybridization and SCFA analysis as described later. Blood samples were centrifuged according to the requirements of the tests to be performed, immediately after blood collection. EDTA-Plasma tubes were transported from the blood collection point to Fondazione Edmund Mach (FEM), plasma was separated by centrifugation at 4000 rpm for 10 min, aliquoted and stored at − 80 °C for further metabolomic and ELISA analysis at FEM laboratories. Blood cells counts, total cholesterol, HDL cholesterol, LDL cholesterol (direct quantification), triglycerides, glucose, insulin, liver and kidney enzymes, homocysteine, folates, C-reactive protein (CRP) were evaluated according to the standard procedures of the Laboratorio di Patologia Clinica of the S. Chiara Hospital, APSS, Trento.

### Plasma lipopolysaccharide (LPS), CD14 and LPS-binding protein (LBP)

Markers of endotoxemia were measured spectrophotometrically using commercial ELISA kits (Enzyme-linked Immunosorbent Assay Kit For LPS, CEB526Ge, Uscn Life Science Inc.; Enzyme-linked Immunosorbent Assay Kit For LBP, SEB406Hu, Uscn Life Science Inc.; CD14 Human ELISA Kit, ab99989, Abcam).

### Urinary F2-isoprostanes

F2-isoprostanes were measured in urine as a marker of oxidant stress, by specific ELISA kit (Urinary Isoprostane ELISA Kit, EA85, Oxford Biomedical Research).

### Urinary and plasma metabolomic profiling and data analysis

Untargeted metabolomics analysis of 24-h urine and plasma samples were performed with high resolution mass spectrometry Orbitrap LTQ-XL (Thermo Fisher, Bremen, Germany), interfaced to a Dionex HPLC system, consisting of an autosampler and quaternary gradient HPLC-pump, as described by Ulaszewska et al. [21]. Samples were randomized for extraction procedure and injection, keeping samples (V1 and V2) belonging to each volunteer within the same batch. Chromatographic and mass spectrometric conditions were as described by Ulaszewska et al. [21, 22]. Specific targeted bile acid analysis in blood samples was performed as described previously by colleagues [23].

The raw files from Orbitrap analysis were converted to mzXML format with the msconvert utility included in ProteoWizard [24]. To eliminate possible instrumental artifacts which can affect Fourier transform mass spectrometers, the raw data were filtered prior to processing by walking through each mass spectra and, starting from the largest peak, eliminating peaks in a 0.3 Da range around the peak. Peaks were only removed if they were below 20% intensity of the larger peak. An R function for the filtering is available in the R package chemhelper [25]. Profiling data were processed with XCMS [24]. R scripts and specific parameters for the pre-processing, statistics as well as the resulting peaklist can be found at https://gitlab.com/metabolomics-analyses/aleurone-urine and https://gitlab.com/metabolomics-analyses/aleurone-plasma. The resulting feature matrix was annotated using CAMERA [26] to group features corresponding to the same parent ion species. To identify the features showing a significant difference between the dietary groups, two independent linear mixed models were fitted to each mass feature. In the first model, the intensity of each feature was modeled as a function of dietary group, the gender of the subject and the individual. Interactions were tested for significance and left out since they were non-significant for all features. In the second model, the effect of dietary group was left out. The comparison of the two models allowed us to identify the features significantly affected by the dietary group factor. The *p* values were obtained using likelihood ratio tests and the collection of *p* values was corrected for multiple testing by controlling the false discovery rate (FDR) and *q*-values calculated [27, 28]. Post hoc multiple comparison tests (uncorrected) was performed to determine specific effects between means and the collection of *p* values corrected as above. Markers contributing to the discrimination between two treatments were identified through a multiple-step procedure as described by Ulaszewska and colleagues [22]. Metabolite profiles are available in “Metabolights” metabolomics repository with accession number MTBLS2902.

### Fecal microbiota analysis

Metataxonomic analysis of fecal microbiota was performed by 16S rRNA amplicon sequencing. Moreover qPCR was used to accurately enumerate fecal bifidobacteria, lactobacilli and total bacteria, to test the potential prebiotic effect of aleurone supplementation. Flow cytometry Fluorescent in situ hybridization (FCM-FISH) was employed as a confirmatory measurement to qPCR to directly enumerate bifidobacteria, lactobacilli and total bacteria in feces without bias that might be introduced by extraction of nucleic acids and PCR.

For FCM-FISH, fecal samples were analyzed as previously described [29]. Extraction of DNA from fecal samples for qPCR and 16S rRNA sequencing analysis was performed using the FastDNA™ SPIN Kit for Feces (MP Biomedicals).

Quantitative PCR amplifications were performed with sets of primers specific for *Bifidobacterium* spp (Bif F: TCG CGT C(C/T)G GTG TGA AAG; Bif R: CCA CAT CCA GC(A/G) TCC AC [29]), *Lactobacillus*/*Leuconostoc*/*Pediococcus* spp (LAB F: AGC AGT AGG GAA TCT TCC A; LAB R: CGC CAC TGG TGT TCY TCC ATA TA [30]), and for total bacteria (Bact 1369: CGG TGA ATA CGT TCC CGG; Prok 1492 TAC GGC TAC CTT GTT ACG ACTT [29]). Reactions were performed at the specified conditions [29], using SsoFAST Evagreen SupemixKit (BIO RAD) and a Lightcycler 480 PCR machine (Roche). Quantifications were done using standard curves obtained by amplifying pure cultures of *Bifidobacterium animalis* BB12 and *Lactobacillus casei* NRRL B-441 (or an in-house FEM strain n.10005), which had been previously quantified by plate counting, respectively, for the relative specific primers. For total bacteria, a mixture of bacterial DNA was obtained by pooling the total fecal genomic DNA from four fecal samples, which had been previously enumerated using FCM.

16S rRNA amplicon sequencing was carried out using the specific bacterial primer set 341F (5´ CCTACGGGNGGCWGCAG 3´) and 806R (5’ GACTACNVGGGTWTCTAATCC 3´) [31] with overhang Illumina adapters, targeting the regions V3 and V4 of 16S rRNA. Total genomic DNA was extracted as described earlier, then it was subjected to PCR amplification by targeting a ~ 460-bp fragment of the 16S rRNA variable region V3–V4. PCR amplification of each sample was carried out using 25 µl reactions with 1 µM of each primer and 12.5 ng of template DNA, using KAPA HiFi HotStart ReadyMix. All PCR amplification was carried out using a GeneAmp PCR System 9700 (Thermo Fisher Scientific) and the following steps—melting step; 94 °C for 5 min (one cycle), annealing step; 95 °C for 30 s, 55 °C for 30 s, 72 °C for 30 s (30 cycles), extension step; 72 °C for 5 min (1 cycle). The PCR products were checked on 1.5% agarose gel and cleaned from free primers and primer dimer using the Agencourt AMPure XP system (Beckman Coulter, Brea, CA, USA) following the manufacturer’s instructions. Subsequently, dual indices and Illumina sequencing adapters Nextera XT Index Primer (Illumina) were attached by 7 cycles PCR (16S Metagenomic Sequencing Library Preparation, Illumina). The final libraries were pooled in an equimolar way in a final amplicon library, after purification by the Agencourt AMPure XP system (Beckman), analysis on a Typestation 2200 platform (Agilent Technologies, Santa Clara, CA, USA), and quantification using the Quant-IT PicoGreen dsDNA assay kit (Thermo Fisher Scientific) by the Synergy2 microplate reader (Biotek). Barcoded library were sequenced on an Illumina® MiSeq (PE300) platform (MiSeq Control Software 2.0.5 and Real-Time Analysis software 1.16.18). Raw sequences were processed with the Dada2 pipeline [32] and the identified amplicon sequence variants (OTUs) were taxonomically assigned with the SILVA database [33]. Alpha diversity was analyzed by comparing different indexes (Chao1, Shannon and number of OTUs) and beta diversity was analyzed through Bray–Curtis dissimilarity index followed by pairwise comparisons using Mann–Whitney U test. Differences in percentage relative abundance were analyzed through Kruskal–Wallis test on bacterial taxa that were present at least at 0.01% in 25% of the samples. The level of significance was set at 0.05%. Raw sequences were submitted to the European Nucleotide Archive (project accession number PRJEB45819).

### SCFA analysis

SCFAs quantification was carried out following the method described elsewhere [34]. The concentrations were calculated by comparing peak areas with those of standards and were expressed as mM concentration in the fecal sample.

### Statistical method

The sample size 34 was determined to detect a change of 0.4 μmol/L total homocysteine (tHcy) in plasma with a standard variation of 0.5 μmol/L (taken from Price et al. [11]) using the Snedecor and Cochran equation with *α* of 0.05 and 1 − *β* of 0.9 [35]. Taking into consideration, the previous study with aleurone in healthy individuals [11], and the need to account for 15% drop-outs, we aimed to recruit 40 individuals per treatment group in the present study*.*

Equality of variance for each parameter was checked through Levene’s test. Differences between treatments were analyzed by comparing the parameters measured at V2 and at V1 (ALV1 vs PLV1, ALV2 vs PLV2, as well as ALV1 vs ALV2, PLV1 vs PLV2,). We also compared the differences V2–V1 (Δ) between the two study groups (ΔAL vs ΔPL). Initial explorative *t* tests and Principal Component Analysis were performed. If differences were observed after paired *t* test, Bonferroni’s FDR corrections were applied and p values were adjusted. Only statistically significant differences (*p* < 0.05) observed after performing the above-mentioned comparisons are reported. For microbiota sequencing analysis and untargeted metabolomics, the statistical tests are described in the respective sections above. Significance was set within the 95% confidence interval. Statistical analysis was performed using the software R [36].

## Results

### Study recruitment and completion

**Fig. 1:**
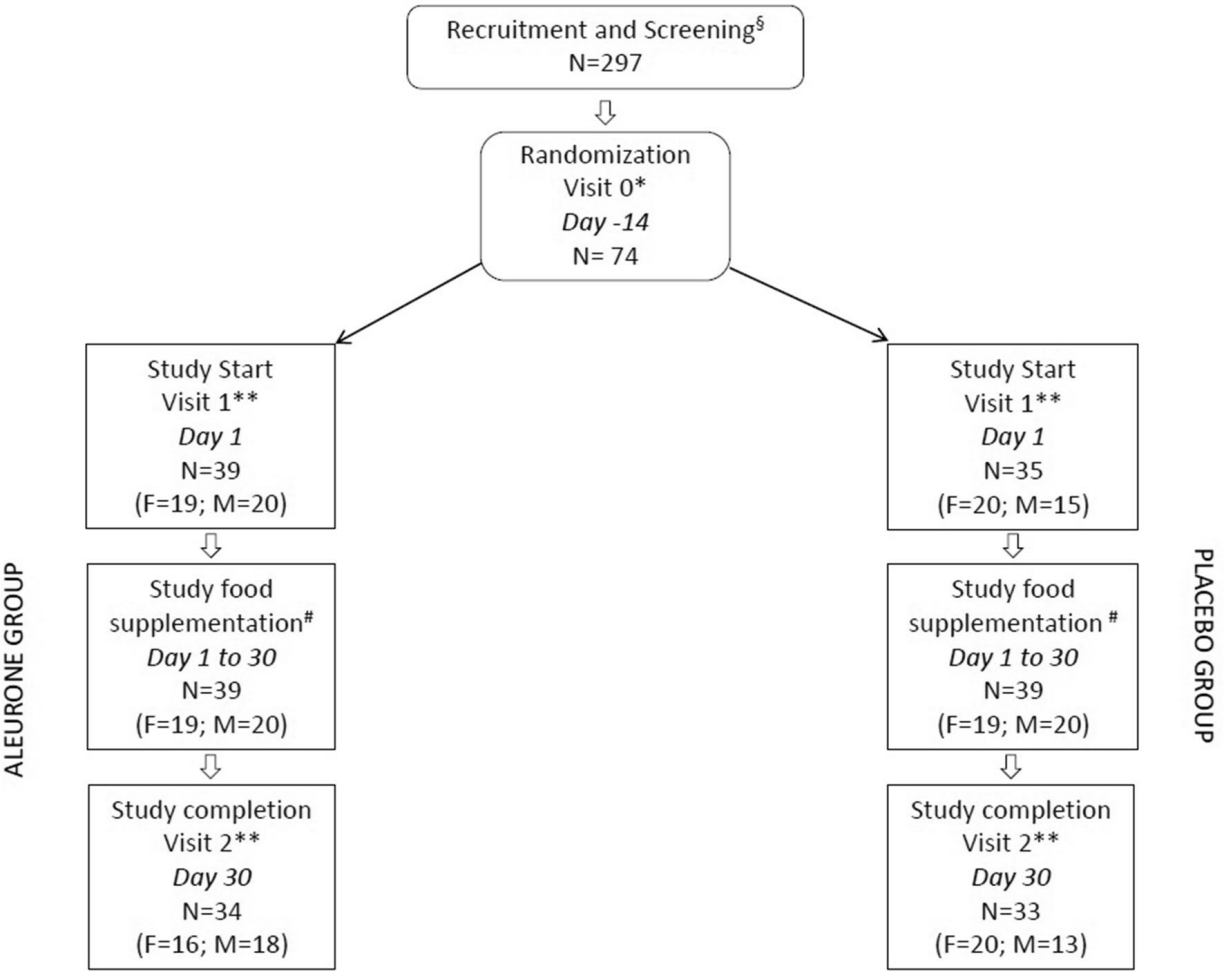
Schematic representation of study design. §Informed consent; Inclusion questionnaire. *Anthropometric and clinical parameters. **Anthropometric and clinical measurements; Biological sample collection; Diet diaries; Intestinal function diaries. # Weekly distribution of study foods

Figure 1 summarizes study design, recruitment and completion. Around 300 volunteers were contacted and interviewed. 74 volunteers were recruited. 67 volunteers, 36 female and 31 male, completed the study. 7 volunteers (8% of the total) dropped out before study completion, 5 of which during dietary supplementation with study product. No adverse events were recorded. Table 2 shows volunteer characteristics and anthropometric measures at the randomization visit (V0). 7 out of 16 women in AL group and 4 out of 20 in PL group were menopausal. 3 volunteers reported to be occasional smokers after being enrolled in the study. Allowed concomitant medications were recorded.

**Table 2:** Characteristics of study participants

	AL	PL
Age (years)	48.1 ± 11.3	46.2 ± 8.3
Wt (kg)	90.8 ± 14.1	90 ± 16.3
Ht (m)	1.7 ± 0.1	1.7 ± 0.1
HR (bpm)	60.4 ± 2.4	60.5 ± 3
SBP (mmHg)	125.8 ± 11.3	122.7 ± 10.4
DBP (mmHg)	77.2 ± 6.6	76.8 ± 5.3
MAP (mmHg)	109.6 ± 8.9	104.6 ± 19
BMI (kg/m^2^)	31 ± 4.9	31.7 ± 4.7
WRC (cm)	17.4 ± 1.2	17.5 ± 1.5
WC (cm)	104.1 ± 11.9	105.1 ± 12.3
HC (cm)	110.7 ± 8.6	112.9 ± 8

Anthropometric and clinical parameters of study participants at randomization, visit V0 (Mean ± SD)

*AL* aleurone-enriched group, *PL* placebo group, *Ht* height, *HR* heart rate, *SBP* sistolic blood pressure, *DBP* diastolic blood pressure, *MAP* median arterial pressure, *BMI* body mass index, *WRC* wrist circumference, *WC* waist circumference, *HC* hips circumference

Statistically significant differences were observed between the two study groups for parameters measured at V2, as described below. No significant differences between treatments were observed when comparing Delta values V2–V1.

### Effect of dietary supplementation on homocysteine levels (primary objective), cardiovascular disease-related biomarkers and dietary intake

After dietary supplementation, the two study groups did not differ in plasma homocysteine levels. Similarly, no significant differences was observed between treatment and placebo group for other anthropometric measures, blood pressure, blood clinical parameters, urinary isoprostane and biomarkers of intestinal permeability (Table 3). Analysis of intestinal bowel habit showed no significant differences in number of evacuations per day, stool consistency nor adverse intestinal effects (data not shown).

**Table 3:** Blood clinical parameters of study participants at recruitment before (V1) and after (V2) dietary supplementation with study foods (mean ± SD)

Blood clinical parameters	AL		PL	
	V1	V2	V1	V2
CRP (mg/L)	2.3 ± 2.5	3.0 ± 3.7	2.9 ± 4.1	3.2 ± 4.2
Glu (mg/dL)	92.6 ± 7.9	91.1 ± 7.1	92.9 ± 9.3	93.6 ± 7.8
TotCHOL (mg/dL)	213.1 ± 44.6	221.1 ± 42.2	216.9 ± 36.4	216.0 ± 35.2
HDLChol (mg/dL)	55.7 ± 13.6	56.3 ± 14.1	56.9 ± 16.5	58.5 ± 17.1
LDLChol (mg/dL)	142.3 ± 33.5	144.5 ± 32.5	141.4 ± 35.3	139.7 ± 34.8
TAG (mg/dL)	138.0 ± 69.2	154.6 ± 94.8	142.2 ± 72.9	138.1 ± 87.4
Homocy s (micromol/L)	15.0 ± 5.3	13.8 ± 4.0	13.1 ± 3.4	12.8 ± 3.9
Folates (ng/mL)	7.5 ± 2.9	7.3 ± 2.2	7.3 ± 2.2	7.2 ± 2.1
Ins (microU/mL)	13.0 ± 6.2	13.7 ± 6.5	13.9 ± 7.4	14.3 ± 6.1
HOMA-IR	3.0 ± 1.4	1.2 ± 1.3	3.3 ± 2.0	1.3 ± 1.4
LPS (pg/ml)	56.8 ± 68.8	53.4 ± 73.4	38.0 ± 21.1	38.3 ± 20.6
CD14 (pg/ml)	153.5 ± 43.8	205.6 ± 153.5	151.7 ± 50.0	156.5 ± 51.2
LBP (pg/ml)	356.4 ± 249.9	391.5 ± 242	326.3 ± 233.7	385.7 ± 223.3
Urinary isoprostane (ng/ml)	0.4 ± 0.4	0.4 ± 0.6	0.5 ± 0.6	0.5 ± 0.7

*AL* aleurone group, *PL* placebo group, *CRP* C Reactive Protein, *Glu* fasting glucose, *TotCHOL* total cholesterol, *HDLChol* high-density lipoprotein cholesterol, *LDLChol* low-density lipoprotein cholesterol, *TAG* Triglycerides, *Homocys* homocysteine, *Ins* Insulin, *HOMA-IR* homeostatic model assessment of insulin resistance, *LPS* lipopolysaccharide, *CD14* cluster of differentiation 14, *LBP* Lipopolysaccharide Binding Protein

Table 4 reports macronutrient intake, after analysis of 3-day food diaries. Significantly, higher intake of dietary fiber was observed at the end of the study compared to baseline for both study groups (18.0 ± 6.2 vs 30.1 ± 8.8, *p* = 0.000019, for AL and 15.7 ± 7.6 vs 31.7 ± 12.7, *p* < 0.0000001, for PL, V1 vs V2). Both AL and PL slightly increased average protein intake at V2 compared to V1, although this was not significantly. However, the placebo cellulose supplemented group showed a significant increase in animal protein at the end of the study compared to the baseline (43.8 ± 19.9 vs 56.4 ± 21.6, *p* = 0.033, for PL, V1 vs V2).

**Table 4:** Participants’ dietary composition after analysis of 3-day food diaries before (V1) and after (V2) dietary supplementation with study foods (mean ± SD)

Macronutrients	AL		PL	
Calories (Kcal)	2168.2 ± 477.0	2320.9 ± 599.7	2178.2 ± 630.0	2100.0 ± 585.9
Proteins (g)	84.0 ± 18.7	97.6 ± 26.6	81.6 ± 26.6	88.8 ± 27.0
Of which animal proteins (g)	47.8 ± 14.5	51.9 ± 22.0	43.8 ± 19.9^a^	56.4 ± 21.6^a^
Carbohydrates (g)	272.5 ± 79.4	272.9 ± 90.6	279.8 ± 98.9	239.6 ± 69.7
Of which sugars (g)	98.5 ± 39.6	87.3 ± 40.0	89.6 ± 38.3	78.5 ± 37.1
Fiber (g)	18.0 ± 6.2^b^	30.1 ± 8.8^b^	15.7 ± 7.6^c^	31.7 ± 12.7^c^
Fats (g)	81.0 ± 23.4	89.9 ± 25.7	81.9 ± 25.6	85.3 ± 34.5
Of which saturated fats (g)	25.7 ± 9.5	30.6 ± 9.5	26.2 ± 10.4	31.1 ± 12.2

Superscript letters indicate significant differences (a: *p* = 0.02; b, c: *p* < 0.01, paired *t* test)

*AL* Aleurone group, *PL* placebo group

### Gut microbiota targeted quantification and 16S rRNA community analysis

Quantitative PCR using specific primers showed a significant increase for *Bifidobacterium* spp for AL at V2 compared to V1 and a significant increase in *Lactobacillus* spp both for AL and PL at V2 compared to V1 (9.71 ± 0.29 vs 9.53 ± 0.4, *p* = 0.02, for AL and 9.69 ± 0.35 vs 9.65 ± 0.36, *p* = 0.5 for PL, Log_10_[*Bifidobacterium* spp/g]; 8.93 ± 0.66 vs 8.52 ± 0.75, *p* = 0.003 for AL, and 8.83 ± 0.82 vs 8.51 ± 0.99, *p* = 0.02 for PL, Log_10_[*Lactobacillus* spp/g]; mean ± SD, V2 vs V1, respectively). Aleurone induced increases in *Bifidobacterium* spp and *Lactobacillus* spp absolute abundances were confirmed using FCM (Supplementary information Fig. 1).

**Fig. 2:**
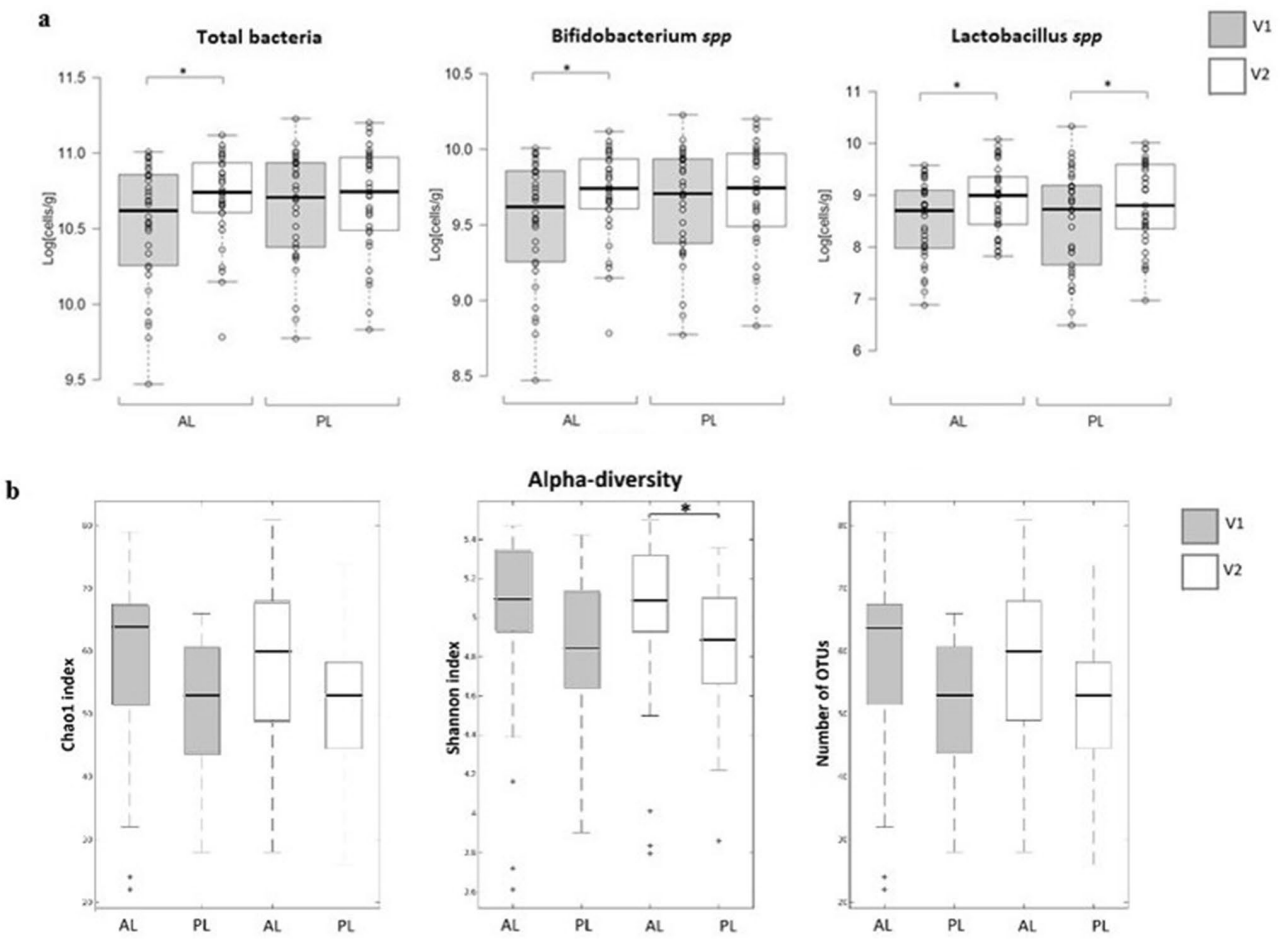

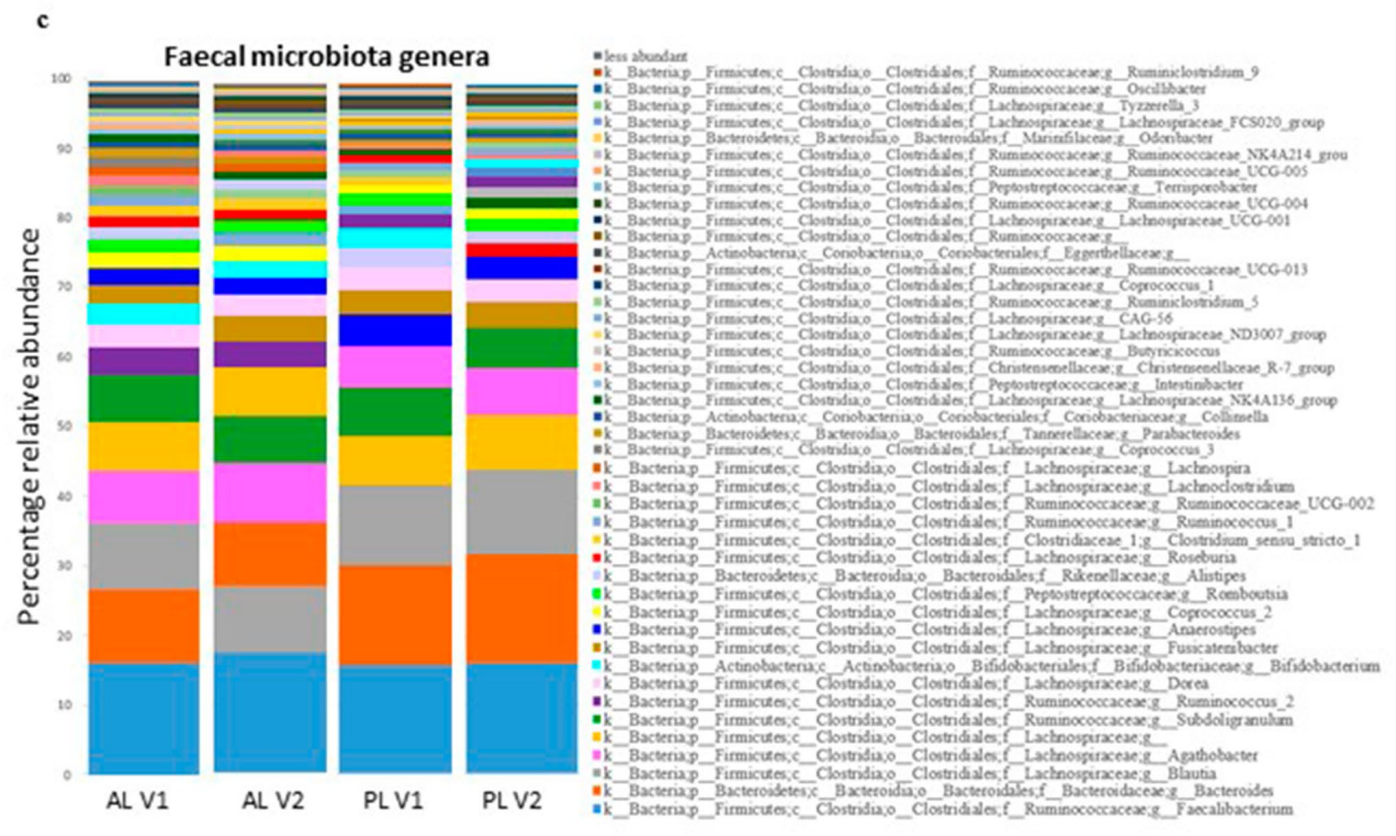

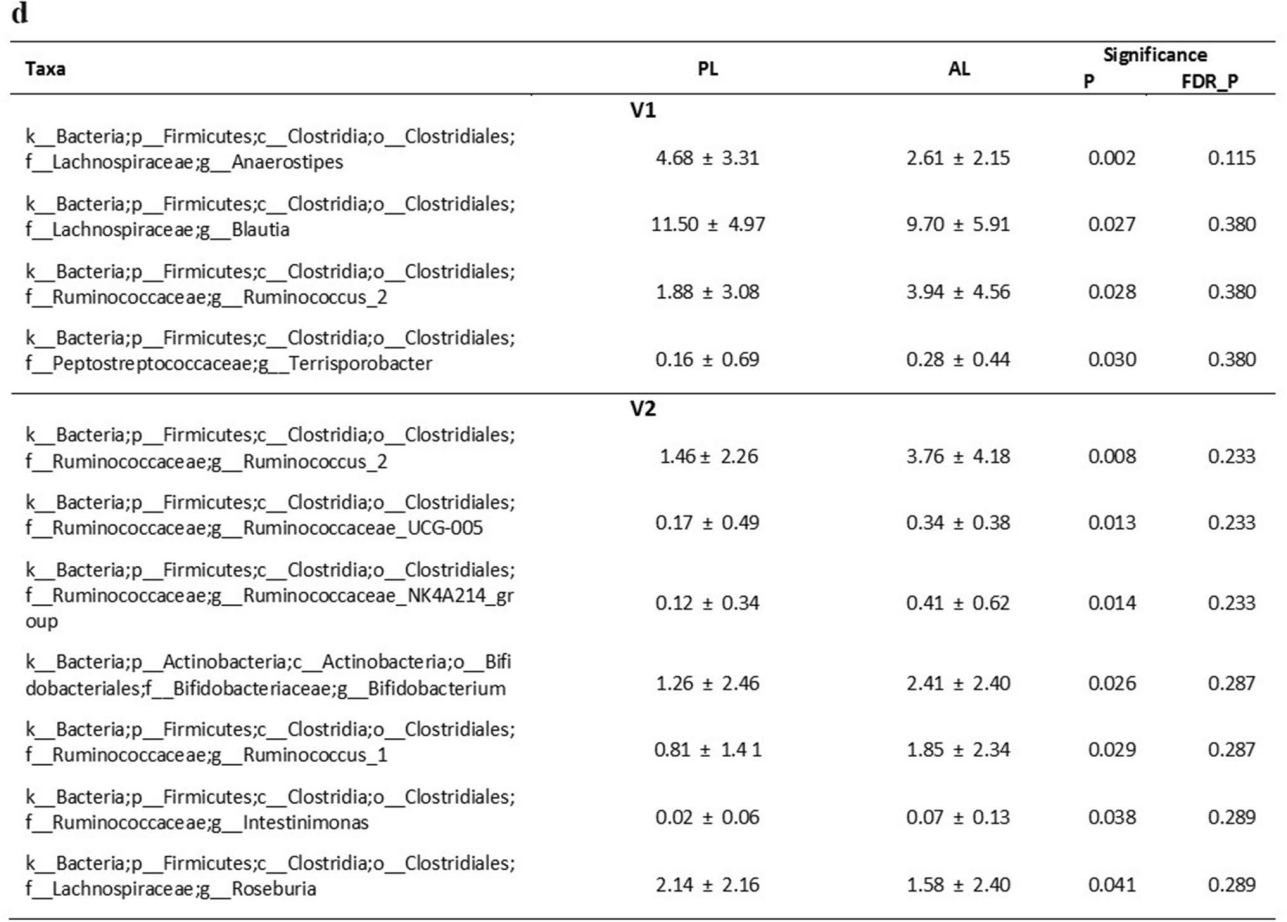
Intestinal microbiota analysis. **a** Enumeration of fecal bacterial groups by quantitative PCR. *p < 0.05 (paired student ‘s *t* test). **B** Alpha diversity plots after V3–V4 16S rRNA sequencing analysis (*p = 0.0138, Mann–Whitney U test with FDR). Center lines of boxplots show the medians; box limits indicate the 25th and 75th percentiles; whiskers extend 15 times the interquartile range from the 25th and 75th percentiles. **c** Histograms of percentage relative abundance of fecal microbiota genera after taxonomic analysis of V3–V4 16S rRNA sequences. *AL* aleurone, *PL* placebo; **d** summary of significant changes in fecal microbiota (Mean ± SD). *P* p value after non parametric Mann–Whitney *U* test, *FDR-P* false discovery rate corrected *p* value. *V1* before dietary supplementation with study foods and *V2* after dietary supplementation with study foods

16S rRNA sequencing produced a total number of 7,994,012 reads. Forward and Reverse sequences were subjected to paired-end read merging, primer trimming and de-noising, thus producing 1,743,254 total number of reads and on average 14,288 reads per sample, corresponding to 1540 OTUs per sample. Maximum number of allowed errors per read was set to two (maxEE = 2) and sequences smaller than 390 bp were removed. Statistical comparison was carried out for those taxa that were present at higher abundance than 0.01% in more than 25% of the samples. Alpha diversity appeared to be slightly higher in AL compared to PL at all timepoints, and at V2, Shannon index was significantly higher in AL compared to PL (Fig. 2B). Beta diversity was significantly different when analyzing Bray Curtis dissimilarity of AL compared to PL at V2. However, few differences in relative abundance of individual taxonomic levels were observed between AL and PL. *Roseburia inulinivorans* species had a significantly higher relative abundance in AL compared to PL at V2 but not at V1 (FDR *p* = 0.0499). Higher relative abundance of *Ruminococcus* and *Bifidobacterium* genera and a lower relative abundance of *Roseburia* and *Bacteroides* genera were observed at V2 in AL compared to PL. However, these differences were not statistically significant after FDR correction.

### SCFA

No significant differences in fecal SCFA were observed between treatments of V2 nor within each treatment (V1 compared to V2) (Table 5).

**Table 5:** Main short chain fatty acids concentration (mmol/L, mean ± SD) before (V1) and after (V2) dietary supplementation

Diet	Time	Acetate	Propionate	Isobutyrate	Butyrate	2-methylbutyrate + isovalerate
AL	V1	109.9 ± 37.4	12.2 ± 6.0	1.5 ± 0.9	12.7 ± 7.3	1.3 ± 1.1
	V2	111.3 ± 38.5	11.9 ± 6.5	1.5 ± 0.5	12.8 ± 8.6	1.2 ± 0.7
PL	V1	108.7 ± 34.3	12.9 ± 6.3	1.4 ± 1.0	13.7 ± 8.3	1.2 ± 1.2
	V2	103.2 ± 34.9	12.8 ± 8.1	1.2 ± 0.7	10.8 ± 5.9	1.0 ± 0.8

2-methylbutyrate and isovalerate were eluted together and quantified as one peak

*AL* aleurone, *PL* placebo

### Untargeted 24-h urine and plasma metabolites and targeted plasma BA

**Fig. 3:**
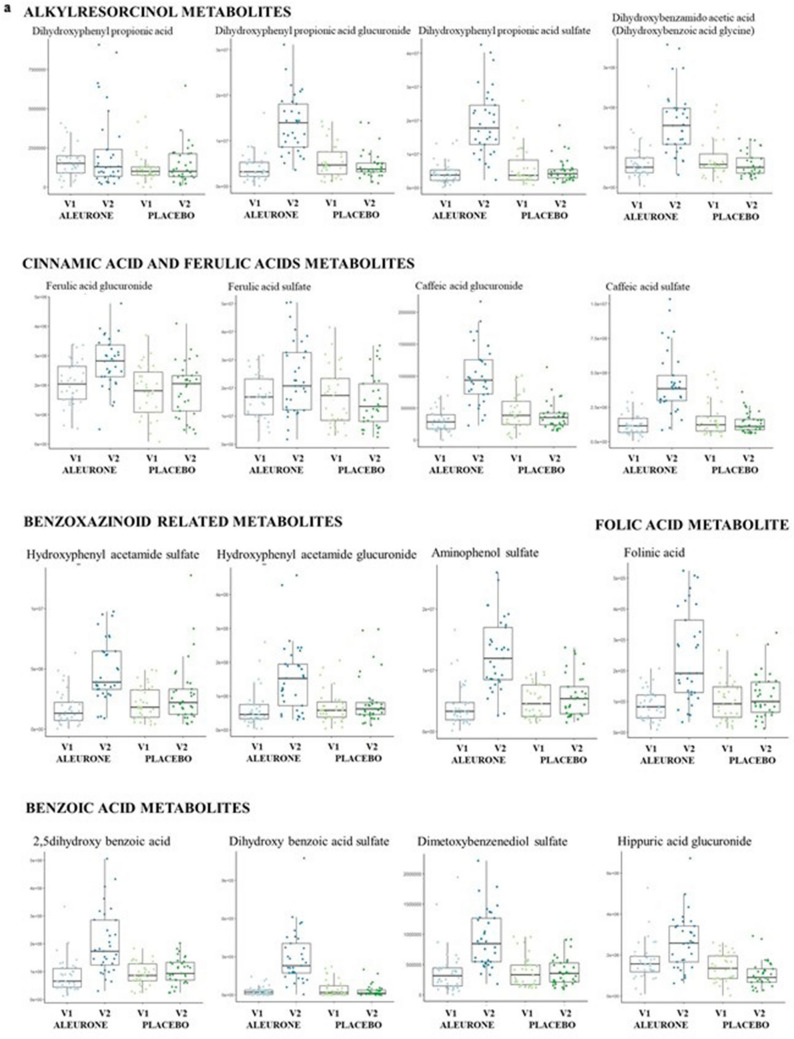

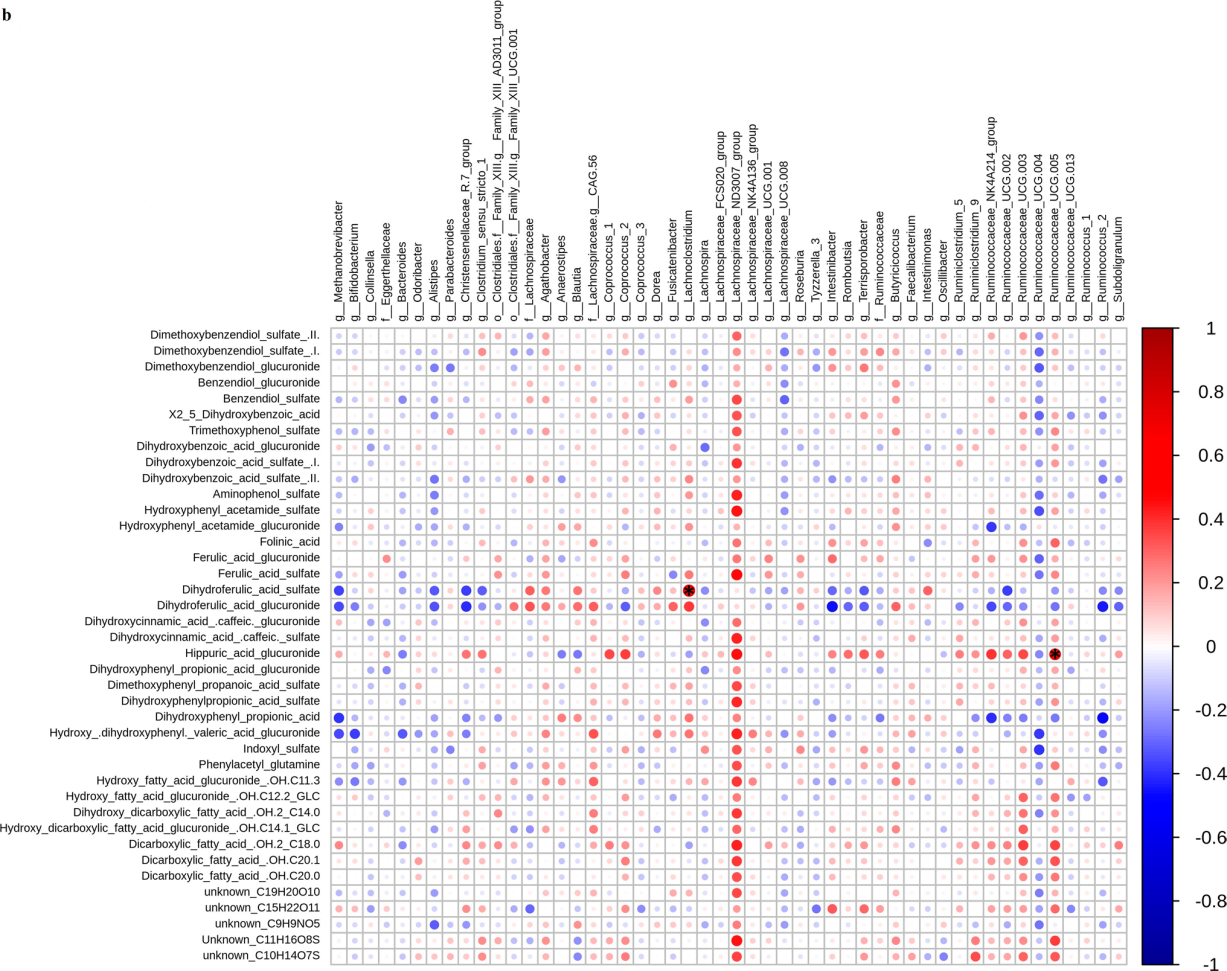

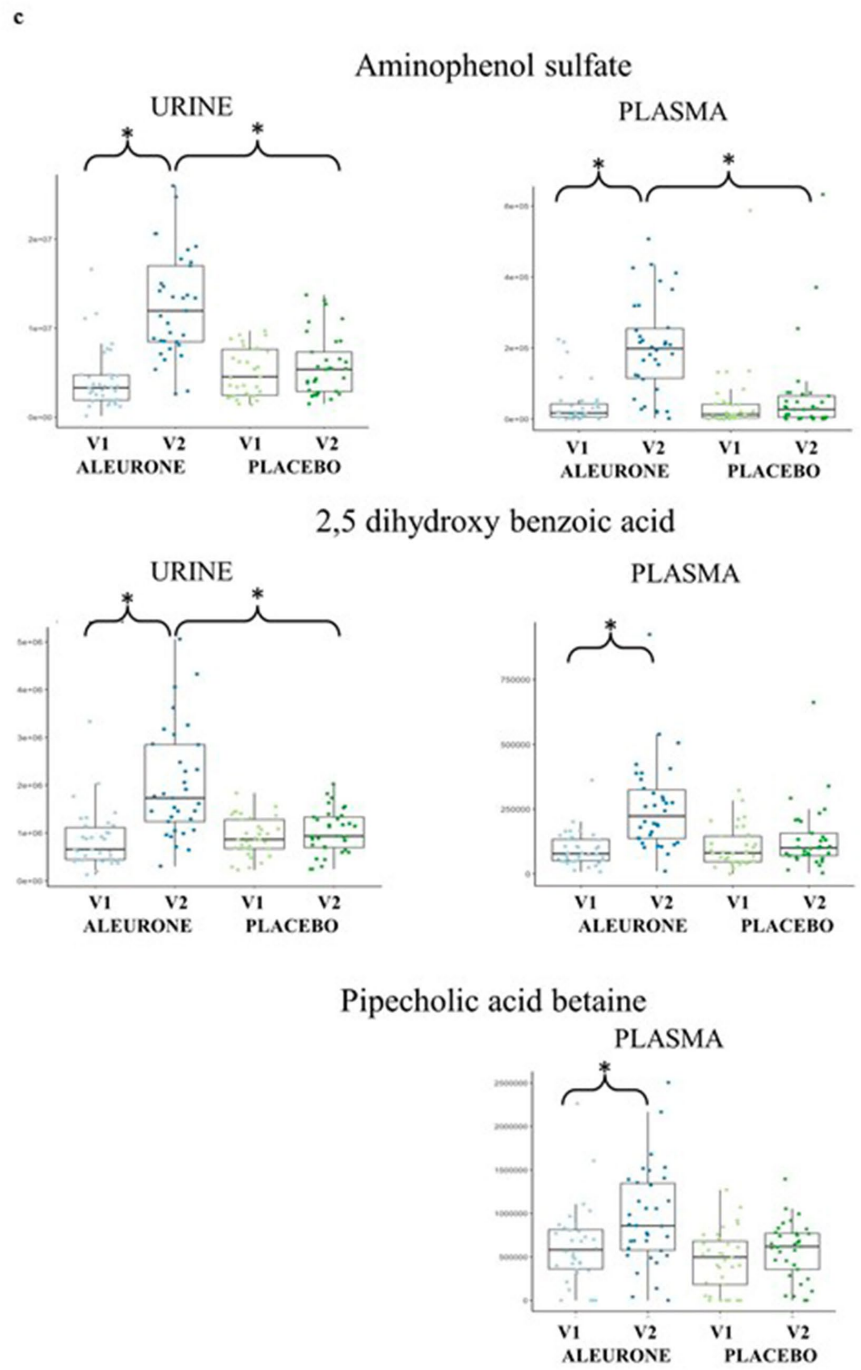

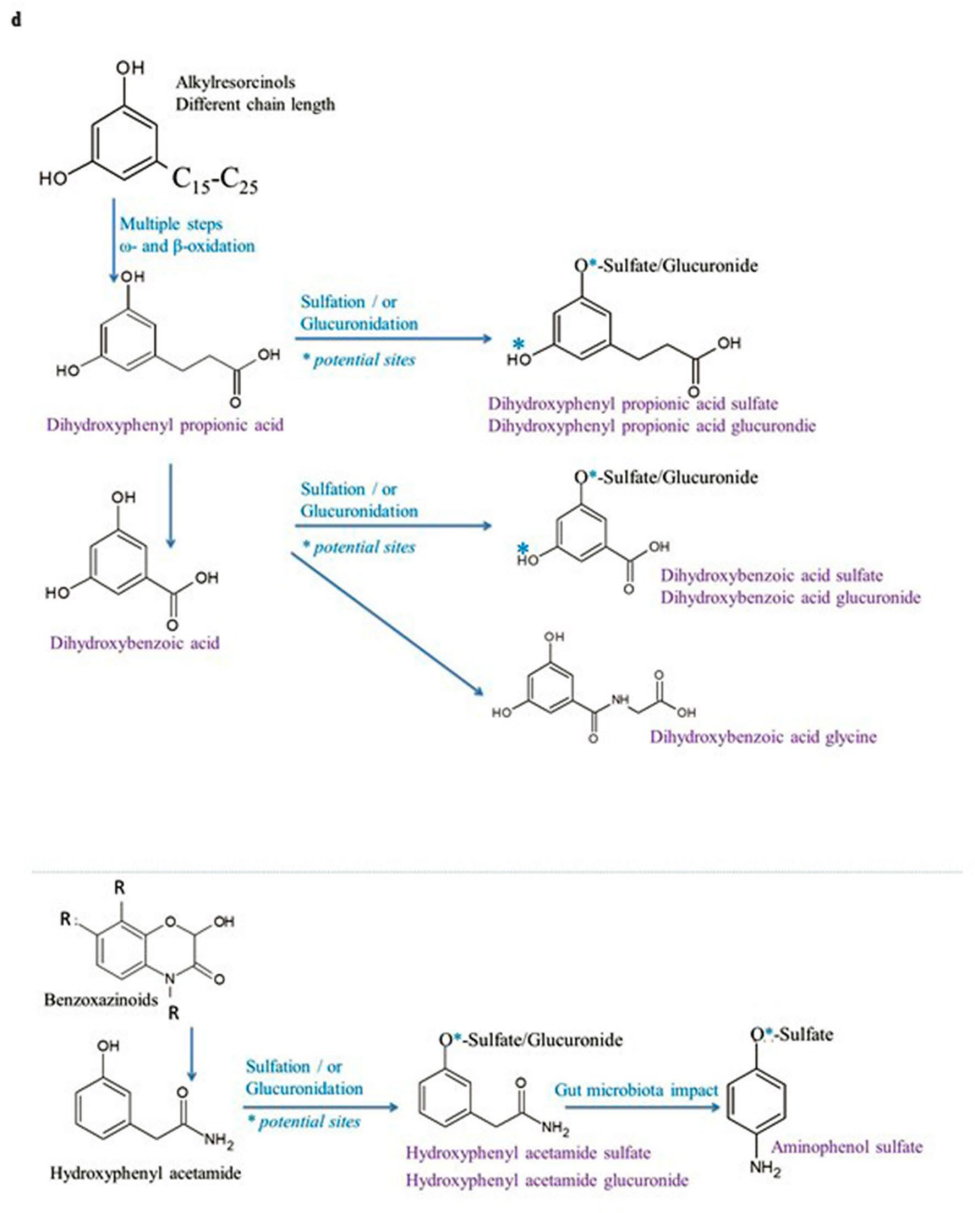
**a** Boxplots showing urinary metabolites that were found significantly higher in urine of AL V2 compared to AL V1 and also compared to PL V2. **b** Correlation heatmap relating urinary metabolites to fecal microbial populations. Dark red indicates strong positive correlation and dark red strong negative correlation (Spearman’s). **c** Boxplots showing metabolites that were significantly different between after aleurone supplementation both in urine and plasma (*P* < 0.01). Center lines of boxplots show the medians; box limits indicate the 25th and 75th percentiles; whiskers extend 15 times the interquartile range from the 25th to 75th percentiles. **d** Alkylresorcinol- and Benzoxazinoid-related metabolites. Fragmentation patterns and adjusted *p* values are reported in Supplementary Information Table 1

Metabolomic analysis revealed significant differences between the two study groups in several metabolites concentration quantified in 24 h urine samples. The number of detected *m/z* features in urine was 8718 and 7875 in negative and positive ionization modes, respectively. In plasma, we detected 3534 and 4829 *m/*z features in negative and positive ionization modes, respectively. Among them, the m/z features discriminating between two dietary interventions were: urine 438 and 218, plasma 11 and 7, respectively, in negative and positive ionization modes. Annotated metabolites were grouped into families based on their chemical similarities: alkylresorcinol metabolites, cinnamic acid and ferulic acids metabolites, benzoic acid metabolites, roasted aroma metabolite, benzoxazinoid-related metabolites, folic acid metabolite, fatty acids metabolites. Nine metabolites remained unknown. Supplementary Information Table 1 reports all annotated and unknown metabolites with their fragmentation patterns and *p* values. Figure 3A shows annotated metabolites that were significantly different between groups at V2 compared to V1. In particular, the following metabolites were significantly higher in the urine of AL after supplementation compared to baseline and also compared to PL: Ferulic acid glucuronide, Ferulic acid sulfate, Hydroxy-(dihydroxyphenyl)-valeric acid, Caffeic acid sulfate, Dihydroferulic acid sulfate, Dihydroferulic acid glucuronide, Dihydroxybenzoic acid, Dihydroxybenzoic acid glucuronide, Aminophenol sulfate, Dihydroxyphenyl propionic acid, Dihydroxyphenylpropionic acid glucuronide, Hydroxy-phenylacetamide glucuronide, Diphenol glucuronide. Aminophenol sulfate was also significantly higher in fasting plasma samples after AL compared to PL and compared to baseline (Fig. 3c). 2,5-dihydroxy benzoic acid and pipecolic acid betaine were significantly higher after AL, compared to baseline, but not compared to PL (Fig. 3c). No other significant changes in metabolite concentrations were observed in fasting blood samples. A complete list of annotated plasma and urine metabolites that were found significantly different between treatments is given in Supplementary Information Table 1. No significant changes were observed in plasma bile acids either over time or between treatments (Supplementary Information Table 2).

### Correlation analysis

**Fig. 4:**
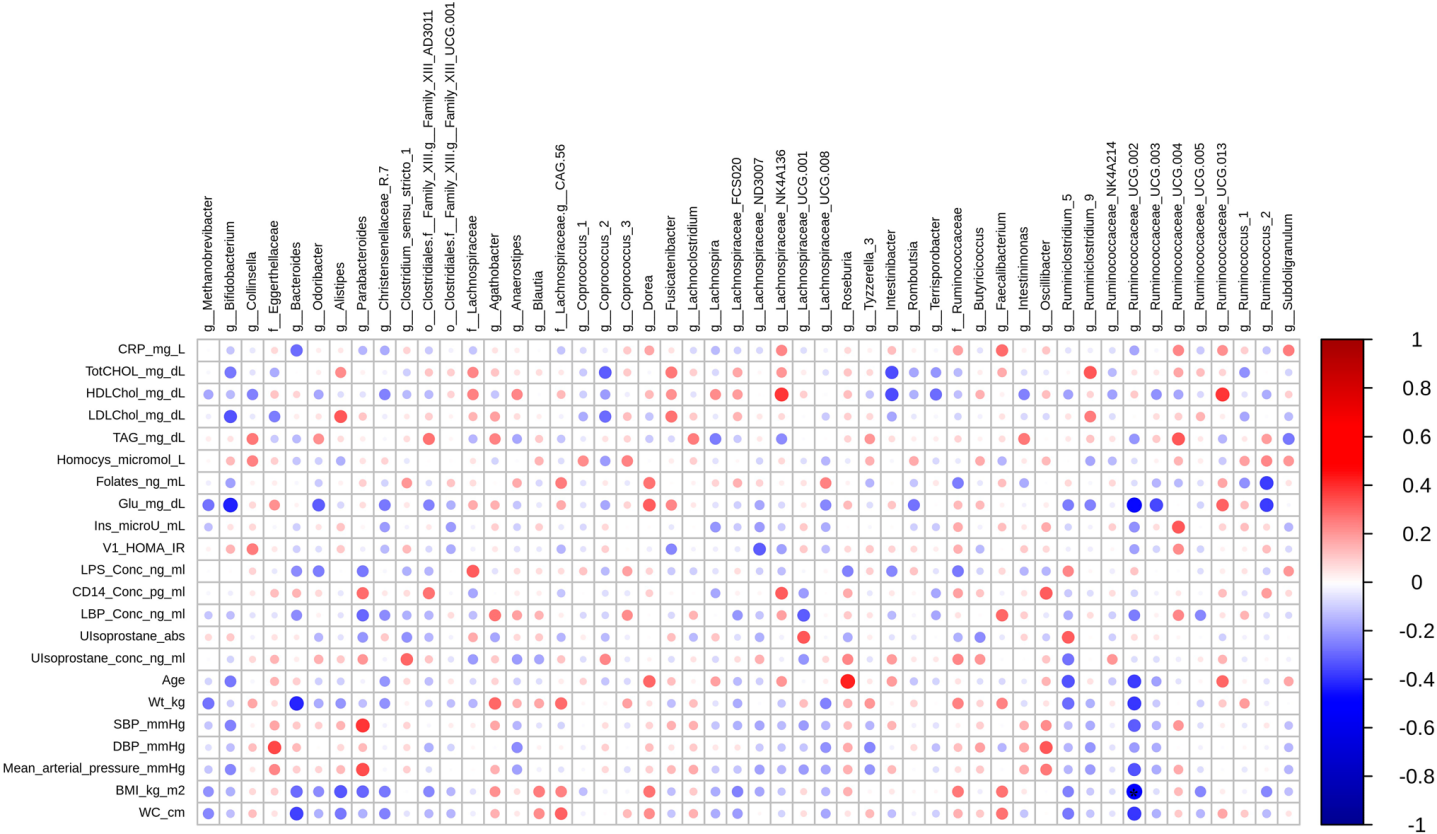
Correlation heatmap relating clinical and anthropometric parameters to fecal microbial populations in the aleurone supplemented group. Dark red indicates strong positive correlation and dark blue strong negative correlation (Spearman’s). *Statistically significant (FDR *p* < 0.05)

Anthropometric and clinical parameters, fecal microbial taxa and urinary and blood metabolites were correlated using Spearman’s correlation. The heatmap in Fig. 3b shows the significant high correlation between urinary dihydroferulic acid sulfate and *Lachnoclostridium* genus (*ρ* = ​0.56, FDR-adjusted *p* = ​0.003), as well as hippuric acid glucuronide and *Ruminococcaceae* UGG005 (*ρ* = ​0.51, FDR-adjusted *p* = ​ 0.015). No other significant correlations were observed after FDR adjustment. Figure 4 highlights the correlations between microbial genera and clinical and anthropometric parameters. A significant inverse correlation was observed between BMI and *Ruminococcaceae* UCG002 (*ρ* = ​− 0.51, FDR-adjusted *p* = 0.02043).

## Discussion

The primary objective of this study was to investigate the effect of aleurone consumption (27 g/day for 4 weeks) on plasma homocysteine concentrations in overweight/obese subjects. Although average plasma homocysteine levels decreased upon AL treatment from 15.0 ± 5.3 to 13.8 ± 4.0 µmol/L, this change was not statistically significant, and homocysteine levels did not differ between AL and PL treatments after intervention. A significant decrease in plasma homocysteine was observed previously by Price and colleagues [11] after chronic supplementation with aleurone. In our study, we employed a less purified form of aleurone, which might have differed from that used by Price et al. [11] in macro- and micronutrient content, including micronutrients related to the homocysteine metabolism. In addition, our study subjects had a higher and more variable concentration of homocysteine, compared with the Price et al. [11] cohort (15 ± 5.3 µmol/L at baseline compared to 9.9 ± 3.1 µmol/L, mean ± SD). Our AL fed study population had a higher age range (48.1 ± 11.3 years compared to 51.5 ± 0.8 years Price et al. [11]) and BMI range (31 ± 4.9 kg/m^2^ compared to 28.7 ± 0.6 kg/m^2^ in the Price et al. study [11]) than the Price et al. study. The more heterogeneous population in our study may have contributed to the lack of statistical significance in AL-induced plasma homocysteine concentrations. It is important therefore, that when measuring the health effects of functional foods, careful consideration be taken when describing the study populations as any observed health effects may be restricted to particular target populations. This is in line with the personalized nutrition concept.

Our secondary objective was to measure the impact of chronic aleurone supplementation on markers of cardiovascular disease risk and on the human gut microbiota and its metabolic output. Aleurone supplementation did not induce statistically significant changes in anthropometric and clinical parameters related to cardiovascular disease risk (Tables 2 and 3). Although increased intake of whole grain cereals is seen as protective against CVD [1, 2], not all studies consistently show improvement in CVD-related parameters. For example, Cicero et al. [39] using a crossover design and 4 week intervention periods, showed that foodstuffs containing ancient wheat (with high levels of non-specific lipid transfer protein 2 as proposed bioactive) could lower blood pressure, triglycerides and fasting blood levels in pre-hypertensive/borderline high pressure subjects (*n* = 63) compared to foodstuffs containing modern wheat varieties low in nsLTP2. Conversely, Vetrani et al. [40] found no effect of whole grain cereal foodstuffs compared to refined grain foodstuffs on glucose homeostasis or plasma lipid parameters in overweight/obese subjects with the metabolic syndrome (*n* = 26) after a 12 week intervention. Heterogeneity in study design, chemical composition of the test foods, lack of clarity on the proposed bioactive involved and differences in dose of bioactive might all contribute to contradictory findings between different studies.

Both dietary fiber and plant polyphenols associated with dietary fiber have been shown to modulate the composition and metabolic activity of the intestinal microbiota [42–44]. Similarly, prebiotics have been shown to increase the abundance of intestinal bifidobacteria and lactobacilli, organisms considered beneficial for human health and often used as probiotics [45]. Based on our previous studies in vitro, we expected modest changes in specific groups of gut bacteria, especially the bifidobacteria, upon AL dietary supplementation [15]. We employed a combined approach using accurate targeted microbiota quantification of bifidobacteria, lactobacilli and total bacteria using qPCR with confirmation by PCR independent FCM-FISH and 16S rRNA targeted probes, and untargeted, community 16S rRNA profiling using partial 16S rRNA gene amplicon sequencing to gain a broader picture of AL-induced changes in microbiome community structure. qPCR showed that aleurone supplementation significantly increased the absolute abundance of *Bifidobacterium* spp after treatment compared to starting levels. Similarly, the absolute abundance of *Bifidobacterium* spp in AL fed subjects was significantly higher than PL fed subjects at V2 after intervention. AL-induced bifidogenesis as determined by qPCR was supported by the FCM-FISH data and was consistent with the 16S rRNA sequencing relative abundance data. However, in the sequencing data, the relative abundance of genus *Bifidobacterium* in the AL group after treatment was not statistically different from the PL fed groups upon FDR correction for multiple testing.

qPCR also highlighted a significant increase in *Lactobacillus* spp for both AL and PL at V2 compared to V1. 16S rRNA sequencing of fecal samples showed alpha-diversity (Shannon diversity index) was higher in the AL group compared to the PL group after treatment and an overall significant change in beta diversity in AL V2 compared to PL V2. Lower relative abundance of *Roseburia inulinivorans* was observed at V2 in AL compared to PL. Higher relative abundance of *Ruminococcus* and *Bifidobacterium* genera and lower relative abundance of *Roseburia* and *Bacteroides* genera were observed in AL compared to PL at V2 (Fig. 3). However, these changes in relative abundance of particular genera were not statistically significant after FDR correction for multiple testing.

In our study volunteers, a significant increase in total dietary fiber intake was observed after supplementation with both the active aleurone and the placebo treatments, and possibly this was due to the amount of dietary fiber contained in study products. Animal protein intake increased after supplementation in the PL fed group. However, protein intake should not influence plasma homocysteine levels [46, 47].

Despite the reported increased intake of fiber, no changes were observed in fecal short chain fatty acids concentrations at the end of the study in both study groups. Reports of changes in fecal SCFA concentrations or profiles following dietary interventions with fibers at moderate intake levels are not consistent across studies [48]. In this present study, the obese volunteers in general appeared to have relatively high levels of fecal acetate compared to published levels, but not of propionate or butyrate levels [49]. Previous studies have also reported that obese individuals may excrete higher quantities of SCFA compared to lean individuals [50, 51]. This result might reflect an increased carbohydrate intake in overweight people, a different absorption mechanism compared to lean people or differences in composition of the gut microbiota present in lean compared to obese individuals. All three are likely to impact on SCFA concentrations in feces. Additionally, this study was of parallel design, with one group fed AL and the other fed PL. A crossover design, where each participant acts as their own control, may have allowed us to overcome some of the interindividual variation in the dataset and identify statistically significant changes in study measures upon AL ingestion.

We measured the concentration of bacterial endotoxin as a marker of intestinal integrity and of metabolic health that may be affected by supplementation with dietary fiber. Intestinal permeability and leakage of LPS across the intestinal mucosa has been associated with the chronic low grade inflammation characteristic of obesity and metabolic disease and thought to trigger insulin resistance [41]. We observed a large inter-individual variation in plasma lipopolysaccharide (LPS) concentrations, as well as LPS soluble receptor CD14 and LPS-binding protein (LBP) across volunteers and these parameters did not change significantly upon AL or PL treatments.

Untargeted 24 h urine metabolite profiling showed a significantly higher amount of aleurone- and whole grain food-derived metabolites in the group fed AL-enriched foods compared to baseline and also compared to the placebo fed group (Fig. 3a, b and Supplementary Information Table 1). Boxplots showing example metabolites per family are reported in Fig. 3a, while their fragmentation spectra details and adjusted *p* values are reported in Supplementary Information Table 1. Some of these metabolites were previously reported as specifically increased after consumption of AL and appear consistently higher in nearly all the volunteers who consumed AL. Some other metabolites that were found in higher concentrations after aleurone intake are also contained in other whole grain foods. All of these metabolites, however, may be considered the products of host:microbiota co-metabolism as microbial bioconversion is necessary in the colon before these compounds appear in blood and subsequently in urine. We chose 24 urine collection to provide a more complete picture of whole body aleurone metabolism, as fasting urine and plasma samples give limited insight into nutrient pharmacokinetics. However, we cannot say definitively whether changes in urinary metabolite concentrations reflect greater excretion or retention of metabolites. A more invasive experimental design, with many postprandial plasma samples and urine collections would be necessary, as performed previously by Trošt et al.[52].

### Alkylresorcinol metabolites

Alkylresorcinols (AR) are phenolic compounds that are present almost exclusively in wheat fiber and rice [53]. Interestingly, AR together with lignans exist mainly in the outer layers of grains, and no ARs are detected in the endosperm [54, 55]. AR are absorbed and thereafter metabolized to 3,5-dihydroxybenzoic acid (DHBA) and 3-(3,5-dihydroxyphenyl)-1-propanoic acid (DHPPA), which have been detected in human urine and plasma [56–58]. It has been suggested that the metabolic pathway of AR is similar to that of tocopherols and is initiated by a cytochrome P450–mediated omega-oxidation of the alkyl side chain, forming hydroxylated AR [56, 59]. They are further oxidized to produce carboxylated AR; subsequently, the side chain of AR is shortened by Beta-oxidation, yielding hydrophilic metabolites. Simplified scheme of the metabolic pathway is depicted in Fig. 3b. We indeed identified five metabolites which we associated directly with AR content of consumed foods, namely: dihydroxyphenyl propanoic acid, dihydroxyphenyl propanoic acid glucuronide, dihydroxyphenyl propanoic acid sulfate and dihydroxybenzamide acetic acid (called also dihydroxybenzoic acid glycine). Our findings are in good agreement with previous studies, as the above-mentioned metabolites have already been proposed as good markers of whole grain wheat intake [24, 60–63].

### Benzoxazinoid-related metabolites

Benzoxazinoids are a group of phytochemicals mostly found in cereal plants. Mature rye grains, thermally processed rye and wheat grains, and their bakery products are the main dietary sources of benzoxazinoids for humans [64]. Interestingly, the amounts of these compounds increases drastically upon the sprouting and hydrothermal processing of the grains before bread making [64]. We found two urinary benzoxazinoids metabolites discriminant for the AL group namely hydroxyphenyl acetamide sulfate and hydroxyphenyl acetamide glucuronide. As reported by Pekkinen et al*.* [65] enzymatically processed AL causes increased excretion of several nitrogen-containing metabolites. Hydroxyphenyl acetamide is a conversion product of benzoxazinoid; however, the gastrointestinal metabolism of benzoxazinoids is not yet fully described. In addition hydroxyphenyl acetamide sulfate can be further degraded by the gut microbiota to aminophenol sulfate, which was also found in our study [66]. According to Pekkinen and colleagues, [65] the aminophenol sulfate was found in urine exclusively after AL or whole grain consumption. Our findings are in good agreement with recent metabolomics studies that have reported an increase in urinary excretion of some benzoxazinoid metabolites in human intervention studies with different types of whole grain breads [67, 68] and after a 5-week dietary intervention with high fiber doses [64].

### Ferulic and cinnamic acids metabolites

Ferulic acid is the most abundant hydroxycinnamic acid in many cereals, and is concentrated in the outermost aleurone layers and bran of the grain [68, 69]. For this reason, it is not surprising that several metabolites belonging to this family were identified in our study. We found 6 metabolites of ferulic and caffeic acid with sulfate and glucuronide patterns being discriminant for AL diet. It must be highlighted that excretion of ferulic acid metabolites suffered from high interindividual variation, while caffeic acid metabolites were less affected. This interindividual variation is likely to be caused by intake of other foods/beverages rich with ferulic acids or its precursor chlorogenic acid such as coffee or tea.

### Benzoic acid metabolites

The high number of benzoic acids metabolites observed in the present study is most likely related to microbial degradation of aleurone constituents. We found benzoic acid metabolites with different hydroxylation patterns, conjugated to sulfate and glucuronide moieties. Notably, we also found benzenediols (i.e. pyrocatechol or resorcinol), most probably the residues of alkyl-resorcinol moieties after the omega-oxidation processes. To our knowledge, no free benzenediols or their sulfate conjugates have been found in urine after aleurone or other whole grain food intake. Hanhineva and colleagues did find, however, glucuronides of nonadecyl-benzenediol and heneicosenyl-benzenediol in urine after whole grain intake [70].

### Folic acid metabolites

In our study, we found folinic acid, a derivative of folic acid, as highly discriminant for AL intake. Aleurone is a rich source of bioavailable folic acid [9]. Fenech and colleagues quantified plasma folate in a randomized, short-term intervention trial with a cross-over design comparing ingestion of 100 g wheat bran cereal (low folate control) versus 100 g AL cereal, and compared to a tablet containing 500 mg folic acid taken together with 100 g wheat bran cereal (high folate control) [10]. The increase in plasma folate over the 7-h period postprandial to AL cereal intake was more than fourfold greater than that observed following the wheat bran cereal, and similar to that observed following the 500 mg folic acid tablet. There is no available evidence that folinic acid could be detected in biological fluids after AL intake, suggesting further studies are required.

Plasma untargeted metabolomics analysis showed only aminophenol sulfate was significantly different between the two intervention groups at V2 (Fig. 3c). 2,5-dihydroxy benzoic acid was found to be significantly higher in urine of ALV2 compared to ALV1 and to PLV2, while for plasma, this compound was higher only in ALV2 compared to ALV1, but not compared to PLV1. This result is to be expected, since, differently from 24 h urine, it is difficult to find host and microbial metabolites of food in fasting plasma samples due to the physiological clearance and absorption processes which happen during the hours immediately following food consumption. To get a clearer picture of how aleurone impacts on the metabolic output of the gut microbiota, specifically designed acute studies measuring the nutri-kinetics of aleurone and derived polyphenolic compounds are warranted.

We quantified different bile acids in plasma, since dietary modulation of bile acid profile might reflect a change in metabolic health. Particularly, we wanted to check whether changes in microbial populations corresponded to changes in bile acid pools. Changes in the diet might affect microbial biotransformation of primary bile acids into secondary and tertiary bile acids and, therefore, modified bile acid pools might differentially affect physiological processes linked to bile acid receptors cascade. However, no changes in total and individual bile acids were observed in fasting plasma samples after intervention. Again this result highlights the importance of acute postprandial sampling during dietary interventions, since fasting plasma might not reflect the physiological changes induced by the consumption of the test food.

Correlation analysis between above-mentioned urine metabolites and microbiota highlighted the role of members of the Ruminococcaceae family in production of hippurate upon aleurone metabolism. Increased hippurate was previously associated with high fruit and whole grain cereal consumption and higher hippurate production has been linked to lower probability of developing metabolic syndrome [71]. Dihydroferulic acid sulfate was observed to be significantly and directly correlated with *Lachnoclostridium* genus, thus indicating a particular involvement of this bacterial taxon in whole grain metabolism [66]. As such, our study confirms the impact of aleurone on the gut microbiota at a metabolic level. Correlating genus level fecal microbiota composition with clinical parameters, several correlations consistent with the literature were observed. For example, inverse relationships between relative abundance of bifidobacteria and fasting glucose and LDL-cholesterol concentrations; Bacteroides relative abundance was negatively associated with body weight, waist circumference and BMI; *Ruminococcus*_UCG-002 relative abundance was also negatively associated with BMI, glucose levels, waist circumference, body weight, age, blood pressure (although these correlations did not hold for other genera of *Ruminococcus*) and Parabacteriodes relative abundance was positively associated with systolic blood pressure and mean arterial pressure.

In conclusion, although we did not observe significant changes in health-related biomarkers upon AL consumption, we did confirm earlier in vitro studies suggesting that aleurone may modulate the gut microbiota in a similar manner to prebiotics. We observed a significant increase in bifidobacteria both over time and compared to the placebo. We have also identified a number of significant and useful biomakers of wheat aleurone intake, all related to wheat polyphenol metabolism by the gut microbiota. Such biomarkers are useful for improving our ability to measure dietary intake and also confirm the important role for the gut microbiota in digestion and metabolism of complex nutrients present in plant foods.

## Supplementary Information

Below is the link to the electronic supplementary material.

Supplementary file1 (DOC 259 KB)

Supplementary file2 (DOC 56 KB)

Supplementary Information Figure.1 Enumeration of faecal bacterial groups before (V1) and after (V2) dietary supplementation as measured by FCM-FISH. AL=aleurone; PL=placebo. *:p<0.05 (FDR corrected paired students’ t test)

## Data Availability

Raw sequencing data obtained for 16S rRNA sequencing of fecal microbiota were registered at European Nucleotide Archive and are publicly available with study accession number PRJEB45819. Metabolomic data were submitted to the MetaboLights EMBL-EBI repository with accession number MTBLS2902.
