# Supplementary material for: Impact of wheat aleurone on biomarkers of cardiovascular disease, gut microbiota and metabolites in adults with high body mass index: a double-blind, placebo-controlled, randomized clinical trial
Source: Eur J Nutr. 2022 Mar 5;61(5):2651–71. doi: 10.1007/s00394-022-02836-9 (PMC9279244; doi:10.1007/s00394-022-02836-9)
Supplement: Supplementary file 1 — Supplementary file1 (DOC 259 KB) [file 394_2022_2836_MOESM1_ESM.doc]

**Supplementary Information**. **Table 1** Annotated metabolites in 24 hours urine and plasma. The table shows metabolites that were significantly different between study groups at the end of treatment and within study groups when comparing baseline with the end of treatment. Adjusted p values for URINE and PLASMA are shown: 1) PLV2 vs ALV2; 2) ALV1 vs ALV2; 3) PLV1 vs PLV2. -: not significant (p>0,01).

| No | Name | **Rt (min)**  **urine/plasma** | **Formula**  **Theo. MW^a^**  **ID Level** | ***m/z* (Relative Intensity)** | **Annotation** | **Polarity** | **MS/MS spectra** | **URINE**  **adj p value:** | **PLASMA**  **adj p value:** | **Comments, references** |
| --- | --- | --- | --- | --- | --- | --- | --- | --- | --- | --- |
| **Alkylresorcinol metabolites** | | | | | | | | | | |
| M1 | Dihydroxyphenyl propanoic acid | 3.95 | C_9_H_10_O_4_  198.0597  Level II | 181.0505 (100)  *182.0538 (8)* | [M-H]^-^ | NEG | **MS/MS^2^ 181:**121.0297(100) [M-H-C_2_H_4_O_2_]-; 137.0609(60)[M-H-CO_2_]-; 163.0406(4) [M-H-H_2_O]- | 1) 2.49E-06  2) 1.3E-03  3)- | - | Metabolomics. 2015;11(1):155-65 |
|  |  |  |  | **183.0650 (100)**  ***184.0683 (9)*** | [M+H]^+^ | POS | **n.a** |  |  |  |
| M2 | (Dihydroxyphenyl) propanoic acid glucuronide | 2.63 | C_15_H_18_O_10_  C_9_H_10_O_4_+GLC  358.0906  Level III | **357.0828 (100)**  ***358.0861 (18)***  ***359.0878 (1)***  **379.0647 (19)**  ***380.0694 (3)***  **395.0384 (8)**  **715.1728 (10)**  ***716.1756 (2)***  181.0506 (15)  *182.0538 (2)* | [M-H]^-^  [M-2H+Na]^-^  [M-2H+K]^-^  [2M-H]^-^  [M-H-GLC]^-^ | NEG | **MS/MS^2^ 329:** 113.0248(100); 175.0247(83)[GLC-H]-;  **MS/MS^3^ 329-181:** 137.0610 (45); 166.0273 (100)  **MS/MS^4^ 329-181-137:** 93.03485 (100)[M-H-C_2_H_4_O]- | 1)5.91E-13  2)5.91E-13  3)- |  | Metabolomics. 2015;11(1):155-65 |
|  |  |  |  | **376.1235 (100)**  ***377.1277 (18)***  **359.0974 (30)**  ***360.1032 (6)***  **165.0544 (16)**  **183.0649 (14)**  ***381.0789 (22)*** | [M+NH_4_]^+^  [M+H]^+^  [M+H-GLC-H_2_O]^+^  [M+H-GLC]^+^  [M+Na]^+^ | POS | **MS/MS^2^ 379:** 183.0650(100); 165.0544(72); 323.0767(20); 359.0977(15); 341.0511 (11);  **MS/MS^3^ 379-183:** 165.0540(100)[M+H-H_2_O]^+^ |  |  |  |
| M3 | Dihydroxyphenyl propanoic acid  Sulfate | 3.19 | C_9_H_10_O_7_S  C_9_H_10_O_4_ +SO_3_  262.0155  Level III | **137.0610 (18)**  ***138.0644 (2)***  **261.0075 (100)**  ***262.0109 (11)***  ***263.0032 (7)***  ***264.0063 (0.8)***  **282.9894 (5)**  **298.9633 (9)**  **523.0220 (5)** | [M-H]^-^  [M-2H+Na]^-^  [M-2H+K]^-^  [2M-H]^-^ | NEG | **MS/MS^2^ 261:**181.0505(100); 135.0453(8); 137.0610(5); 96.9597(4)  **MS/MS^3^ 261-181:** 137.0609(100)  **MS/MS^4^ 261-181-137:** 95.0505(100) | 1)5.91E-13  2)5.91E-13  3)- | - | Metabolomics. 2015;11(1):155-65 |
|  |  |  |  | **280.0483 (100)**  ***281.0516 (11)***  **297.0749 (30)**  263.0218 (8) | [M+NH_4_]^+^  [M-H+NH_4_]^+^  [M+H]^+^ | POS | **n.a** |  |  |  |
| M4 | Dihydroxybenzamido  acetic acid  (dihydroxybenzoic acid glycine) | 2.10 | C_9_H_9_NO_5_  211.0481  Level III | **210.0408 (100)**  ***211.0442 (11)*** | [M-H]^-^ | NEG | **MS/MS^2^ 210:** 192.0300 (40) [M-H-H_2_O]^-^; 166.0511 (100) [M-H-CO_2_]^-^ | 1) 5.91E-13  2) 5.91E-13  3) - | - | doi:10.3945/jn.113.184663 |
|  |  |  |  | 212.0551 (100)  *213.0585 (11)* | [M+H]^+^ | POS | n.a |  |  |  |
| **Cinnamic acid and ferulic acids metabolites** | | | | | | | | | | |
| M5 | Ferulic acid glucuronide | 4.20 | C_16_H_18_O_10_  C_10_H_10_O_4_+GLC  370.0900  Level II | **369.0824 (100)**  **370.0857 (19)**  **193.0506 (36)** | [M-H]^-^ | NEG | **MS/MS^2^ 369:** 175.0249(95)[GLC-H]-; 193.0505(64)[M-H-GLC]-; 113.0246(100)  **MS/MS^3^ 369-193:** 149.0608(100); 178.0273(77); 134.0374(30) | 1) 1.79E-02  2) 1.88E-04  3) - |  |  |
|  |  |  |  | n.a | n.a | POS | n.a |  |  |  |
| M6 | Ferulic acid sulfate | 4.53 | C_10_H_10_O_7_S  C_10_H_10_O_4_+SO_3_  274.0154  LeveL II | **273.0068 (100)**  ***274.0103 (14)***  193.0505 (30)  *194.0539 (3)* | [M-H]^-^  [M-H-SO_3_]^-^ |  | **MS/MS^2^ 273:** 193.0507(100) [M-H-SO_3_]^-^; 229.0176(15)[M-H-CO_2_]-; 96.9604(5); 149.0609(3);  **MS/MS^3^ 273-193:** 149.0608(100); 178.0273(77); 134.0374(30) | 1) 2.05E-02  2) 9.67E-03  3) |  | doi: 10.1186/1743-7075-11-1 |
|  |  |  |  | n.a | n.a | POS | n.a |  |  |  |
| M7 | Dihydroferulic acid sulfate | 4.36 | C_10_H_12_O_7_S 276.031  Level II | **195.0662 (30)**  *196.0696 (4)*  **275.0228 (100)**  ***276.0263 (13)***  ***277.0186 (8)***  *278.0216 (1)*  **297.0048 (28)**  **573.0345 (18)** | [M-H-SO_3_]^-^  [M-H]^-^  [M-2H+Na]^-^  [2M-2H+Na]^-^ | NEG | **MS/MS^2^ 275:** 195.0662(100) [M-H-SO_3_]^-^; 149.0610(14)  **MS/MS^3^ 275-195:** 151.0765(100); 136.0530(95); 135.0453(60); 177.0557(22); 123.0452(18); | 1)2.05E-02  2)-  3)- | - |  |
|  |  |  |  | n.a | n.a | POS | n.a |  |  |  |
| M8 | Dihydroferulic acid glucuronide | 4.37 | C_16_H_20_O_10_  372.1056  Level II | 371.0979(100)  *372.1013 (19)*  *373.1033 (3)*  *195.0660 (13)* | [M-H]^-^  [M-H-GLC]^-^ | NEG | **MS/MS^2^ 371:** 195.0661(82);113.0246(86); 175.0248(57);  353.0876(44); 371.0983(13); 309.0979(6); 119.0504(5);  **MS/MS^3^ 371-195:** 177.0557(100);119.0507(20) | 1)2.5E-02  2)-  3)- | - | Metabolomics. 2015;11(1):155-6 |
|  |  |  |  | **390.1392 (100)**  ***391.1429 (18)***  *392.1443 (4)*  **395.0944 (6)** | [M+NH_4_]^+^  [M+Na]^+^ | POS | **MS/MS^2^ 390:** 179.0699(100); 139.0595 (30) |  |  |  |
| M9 | Dihydroxycinnamic acid glucuronide  (caffeic acid glucuronide) | 3.12 | C_15_H_16_O_10_  C_9_H_8_O_4_+GLC  356.0743  Level II | **355.0661 (100)**  ***356.0652(12)***  **179.0350 (18)** | [M-H]^-^  [M-H-GLC]^-^ | NEG | **MS/MS^2^ 355:**179.0350(100) | 1) 5.91E-13  2) 5.91E-13  3)- |  |  |
|  |  |  |  | 374.1079 | [M+NH_4_]^+^ | POS | **n.a** |  |  |  |
| M10 | Dihydroxycinnamic acid (Caffeic acid) sulfate | 3.91 | C_9_H_8_O_7_S  C_9_H_8_O_4_+SO_3_  259.9991  Level II | **258.9918 (100)**  *259.9943 (13)*  *260.9898 (9)*  **137.0610 (25)**  **179.0350 (13)** | [M-H]^-^  [M-H-SO_3_]^-^ | NEG | **MS/MS^2^ 258:** 179.0350 (100);  **MS/MS^3^ 258-179:** 137.0609 (100) | 1) 6.16E-05  2) 5.91E-13  3)- |  | doi: 10.1186/1743-7075-11-1 |
|  |  |  |  | n.a | n.a | POS | n.a. |  |  |  |
| M11 | Dimethoxyphenyl propanoic acid sulfate | 5.03 | C_11_H_14_O_7_S  C_11_H_14_O_4_+SO_3_  290.0460  Level III | **289.0386 (100)**  ***290.0421 (14)***  209.0818 (22)  *210.0851 (3)* | [M-H]^-^  [M-H-SO_3_]^-^ | NEG | **MS/MS^3^ 289:** 209.0820 (100) [M-H-SO_3_]^-^  **MS/MS^3^ 289-209:** 194.0581(100)[M-H-CH_3_]-; 165.0920(16)[M-H-CO_2_]-; 133.0658(11); 149.0605(9) [M-H-C_2_H_4_O_2_]-; 123.0454(8) [M-H-C_4_H_6_O_2_]-; 135.0446(6) [M-H-C_3_H_6_O_2_]-; 177.0555(5) [M-H-CH_2_-H_2_O]-; | 1) 8.69E-05  2) 9.19E-10  3) - |  |  |
|  |  |  |  | n.a | n.a | POS | n.a |  |  |  |
| **Benzoic acid metabolites** | | | | | | | | | | |
| M12 | Benzenediol glucuronide | 1.30 | C_12_H_14_O_8_  C_6_H_6_O_2_+GLC  286.0689  Level II | **285.0613 (100)**  ***286.0647 (12)***  **571.1298 (10)**  109.0298 (5) | [M-H]^-^  [2M-H]^-^ | NEG | **MS/MS^2^ 285:** 113.0246(100); 175.0248(53)[GLC-H]^-^; 85.0297(15); 95.0141(14); 267.0503(10) [M-H-H_2_O]^-^;  **MS/MS^2^ 285-109:** 73.0190 (100) [M-H-2xH_2_O]^-^; | 1) 8.69E-05  2) 2.36E-12  3) - |  |  |
|  |  |  |  | **309.0579 (100)**  ***310.0613 (12)***  **304.1026 (25)** | [M+Na]^+^  [M+NH_4_]^+^ | POS | **n.a** |  |  |  |
| M13 | Benzenediol  sulfate | 1.44 | C_6_H_6_O_5_S  C_6_H_6_O_2_ +SO_3_  189.9935  Level II | **188.9862 (100)**  ***189.9895 (8)***  **109.0297 (18)** | [M-H]^-^  [M-H-SO_3_]^-^ | NEG | **MS/MS^2^ 188:** 109.0297(100) [M-H-SO_3_]^-^; 125.0245(33) | 1)8.71E-03  2) 9.44E-07  3)- |  |  |
|  |  |  |  | n.a | n.a | POS | **n.a** |  |  |  |
| M14 | Dimethoxybenzenediol  sulfate (I) | 2.60 | C_8_H_10_O_7_S  C_8_H_10_O_4_ +SO_3_  250.0147  Level III | **249.0075 (100)**  *250.0106 (11)*  230.9967 (80)  *231.9998 (8)* | [M-H]^-^  [M-H-H2O]^-^ | NEG | **MS/MS^2^ 249:** 169.0508 (100); 185.0457 (20); 154.0273 (10)[M-H-CH4]; 153.0193 (13)[M-H-CH3]; 79.9575 (5)  **S/MS^3^ 345-169:** 154.0273 (100) | 1) 1.80E-03  2) 6.91E-07  3) - |  |  |
|  |  |  |  | n.a | n.a | POS | n.a |  |  |  |
| M15 | Dimethoxybenzenediol  sulfate (II) | 3.56 | C_8_H_10_O_7_S  C_8_H_10_O_4_+SO_3_  250.0147  Level III | **249.0075 (100)**  *250.0106 (11)*  230.9967 (80)  *231.9998 (8)* | [M-H]^-^  [M-H-H2O]^-^ | NEG | **MS/MS^2^ 249:** 169.0508 (100); 185.0457 (20); 154.0273 (10)[M-H-CH4]; 153.0193 (13)[M-H-CH3]; 79.9575 (5)  **S/MS^3^ 345-169:** 154.0273 (100) | 1) 5.13E-06  2) 3.10E-09  3) - |  |  |
|  |  |  |  | n.a | n.a | POS | n.a |  |  |  |
| M16 | Dimethoxybenzenediol  glucuronide | 2.23 | C_14_H_18_O_10_  C_8_H_10_O_4_+GLC  346.0900  Level III | **345.0826 (100)**  ***346.0860 (16)*** | [M-H]^-^ | NEG | **MS/MS^3^ 345:** 327.0725 (100); 175.0250 (50); 113.0247(80); 169.0508 (30)  **MS/MS^3^ 345-169:** 154.0273 (100)[M-H-CH_3_]- | 1) 9.67E-03  2) 8.71E-03  3) - |  |  |
|  |  |  |  | 364.1237(100)  *347.0973 (80)* | [M+NH_4_]^+^  [M+H]^+^ | POS | **MS/MS^3^ 347:** 329.0713 (100); 301.0760 (15) |  |  |  |
| M17 | 2,5 dihydroxy benzoic acid | U 4.67/ P 4.90 | C_7_H_6_O_4_  154.0266  Level I | **153.0195 (100)**  ***154.0229 (10)***  **109.0297 (16)**  **135.0089 (19)** | [M-H]^-^ | NEG | **MS/MS^2^ 153:** 135.0089(100); 109.0300(70); | 1) 5.13E-06  2) 5.91E-13  3) - | 1) -  2) 2.30E-05  3) - |  |
|  |  |  |  | n.a | n.a | POS | n.a |  |  |  |
| M18 | Dihydroxybenzoic acid sulfate (I) | 2.05 | C_7_H_6_O_7_S  C_7_H_6_O_4_+SO_3_  233.9834  Level II | 232.9760 (100)  *233.9793 (9)*  ***234.9781 (6)***  **254.9579 (12)**  ***255.9612 (8)***  153.0194 (15)  *154.0227 (2)*  **109.0409 (12)** | [M-H]^-^  [M-2H+Na]^-^  [M-H-SO_3_]^-^ | NEG | **MS/MS^2^ 232:** 153.0175(100); 188.9865(50); 85.0821(12)  **MS/MS^2^ 232-153:** 109.0296(100) | 1) 5.91E-13  2) 5.91E-13  3) - |  | doi: 10.1186/1743-7075-11-1 |
|  |  |  |  | n.a | n.a | POS | **n.a** |  |  |  |
| M19 | Dihydroxybenzoic acid sulfate (II) | 2.59 | C_7_H_6_O_7_S  C_7_H_6_O_4_+SO_3_  233.9834  Level II | **232.9760 (100)**  ***233.9793 (9)***  ***234.9781 (6)***  **153.0194 (15)**  ***154.0227 (2)***  204.9814 (80)  *205.9847 (6)*  *206.9772 (4)* | [M-H]^-^  [M-H-SO_3_]^-^  [M-H-CO]^-^ | NEG | **MS/MS^2^ 232:** 153.0175(100); 188.9865(50); 85.0821(12)  **MS/MS^2^ 232-153:** 109.0296(100) | 1) 8.71E-03  2) 4.44E-04  3) |  | doi: 10.1186/1743-7075-11-1 |
|  |  |  |  | n.a | n.a | POS | **n.a** |  |  |  |
| M20 | Dihydroxybenzoic acid glucuronide | 1.45 | C_13_H_14_O_10_  C_7_H_6_O_4_+GLC  330.0587  Level II | **329.0511 (100)**  ***330.0545 (15)*** | [M-H]^-^ | NEG | **MS/MS^2^ 329:** 113.0248(100); 175.0247(83)[GLC-H]-; 153.0192(40)[M-H-GLC]^-^ | 1) 5.91E-13  2) 5.91E-13  3)- |  |  |
|  |  |  |  | **348.0923 (100)**  *349.0963 (16)*  **353.0485 (90)**  *354.0519 (14)* | [M+NH_4_]^+^  [M+Na]^+^ | POS | **n.a** |  |  |  |
| M21 | Trimethoxyphenol sulfate | 4.80 | C_9_H_12_O_7_S  C_9_H_12_O_4_+SO_3_  264.0304  Level III | **263.0229 (100)**  ***264.0263 (12)***  **168.0429 (15)** | [M-H]^-^ | NEG | **MS/MS^2^ 263:** 247.9996 (100); 183.0663 (5)[M-H-SO3]-; 168.0430 (4) [M-H-SO_3_-CH_3_]-; 145.0618 (3); 79.9576 (11) | 1) 2.27E-11  2) 5.91E-13  3) - |  |  |
|  |  |  |  | - | [M+H]^+^ | POS | **-** |  |  |  |
| M22 | Hippuric acid glucuronide | 3.81 | C_15_H_17_NO_9_  C_9_H_9_NO_3_+GLC  355.0903  Level II | **354.0829 (100)**  ***355.0862 (18)***  **376.0643 (12)**  **178.0508 (30)** | [M-H]^-^  [M-2H+Na]^-^  [M-H-GLC]^-^ | NEG | **MS/MS^2^ 354:** 178.0508(100); 175.0247(40); 113.0246(20)  **MS/MS^3^ 354-178:** 134.0612(100) | 1) 1.11E-06  2) 8.69E-05  3)- |  |  |
|  |  |  |  | **378.0793 (30)**  *379.0826 (18)*  **373.1239 (100)**  *374.1272 (18)* | [M+Na]^+^  [M+NH_4_]^+^ | POS |  |  |  |  |
| **Benzoxazinoid related metabolites** | | | | | | | | | | |
| M23 | Hydroxyphenyl acetamide sulfate | 3.77 | C_8_H_9_NO_5_S  C_8_H_9_NO_2_+SO_3_  231.0201  Level III | 150.0563 (100)  *151.0596 (10)*  **108.0468 (30)**  *109.0492 (9)*  **230.0128 (30)**  **231.0161 (5)**  **232.0085 (3)** | [M-H-SO_3_]^-^  [M-H-SO_3_-CO_2_]^-^  [M-H]^-^ | NEG | **MS/MS^2^ 230:** 150.0561(100) [M-H-SO_3_]^-^; 96.9603(27)[HSO_4_]^-^; 108.0455(9) [M-H-SO_3_-CO_2_]^-^;  **MS/MS^3^ 230-150:** 108.0457(100) | 1) 2.25E-06  2) 5.91E-13  - | - | Metabolomics2015;11(1):155-65  doi: 10.1186/1743-7075-11-1  doi:10.3945/jn.114.196220 |
|  |  |  |  | n.a | n.a | POS | n.a |  |  |  |
| M24 | Hydroxyphenyl acetamide glucuronide | 4.04 | C_14_H_17_NO_8_  C_8_H_9_NO_2_+GLC  327.0954  Level III | **326.0878 (100)** | [M-H]^-^ | NEG | n.a | 1) 6.0E-04  2) 5.31E-05  3)- | - | Metabolomics2015;11(1):155-65  doi: 10.1186/1743-7075-11-1  doi:10.3945/jn.114.196220 |
|  |  |  |  | **328.1025 (100)**  ***329.1059 (19)***  **152.0704 (30)** | [M+H]^+^  [M+H-GLC]^+^ | POS | **MS/MS^2^ 328:** 152.0704(100); 110.0598(10); 292.0813(16); 310.0921(8); 134.0598(4)  **MS/MS^3^ 328-152:**110.0598(100); 124.0391(4); 108.0440(4) |  |  |  |
| M25 | Aminophenol sulfate | 1.43/  1.53 | C_6_H_7_NO_4_S  C_6_H_7_NO+SO_3_  189.0096  Level III | **188.0024 (100)**  *189.0045 (8)*  *199.9992 (6)*  108.0458 (33) | [M-H]^-^  [M-H-SO_3_]^-^ | NEG | **MS/MS^2^ 188:** 108.0457 (100) [M-H-SO_3_]^-^ | 1) 5.91E-13  2) 5.91E-13  3) - | 1) 5.19E-04  2) 7.13E-09  3)- |  |
|  |  |  |  | **190.0167 (100)**  *191.0201 (9)*  **227.9726 (26)**  207.0432 (8) | [M+H]^+^  [M+Na]^+^  [M+NH_4_]^+^ | POS | **MS/MS^2^ 190:** 126.0548(100)[M+H-SO_2_]-; 108.0442(30) [M+H-H_2_SO_3_]- |  |  |  |
| **Folic acid metabolite** | | | | | | | | | | |
| M25 | Folinic acid  (5-Formyl-5,6,7,8-tetrahydrofolic acid) | 3.66 | C_20_H_23_N_7_O_7_  473.1659  Level I | **472.1583 (100)**  ***473.1622 (20)*** | [M-H]^-^ | NEG | **MS/MS^2^ 472:**404.1573 (100); 343.1159 (38); 300.1104 (65); 454.1493 (5) | 1) 1.42E-05  2) 5.91E-13  3) - |  | analytical standard |
|  |  |  |  | 474.1726 (100)  *475.1755 (20)*  **327.3611 (14)** | [M+H]^+^ | POS | **MS/MS^2^ 474:** 327.1198 (100); 345.1303 (75); 456.1621 (20) |  |  |  |
| **Others** | | | | | | | | | | |
| M26 | Phenylacetyl-glutamine | 4.55 | C_13_H_16_N_2_O_4_  264.1110  Level I | 263.1033 (100)  *264.1064 (15)*  *265.1087 (1.2)*  **623.2084 (10)**  **624.2120 (3)** | [M-H]^-^  [2M+N6HCl adduct]^-^ | NEG | **MS/MS^2^ 623:** 359.0977 (100)  **MS/MS^2^ 263:** 145.0620 (100); 245.0932 (15); 127.0516 (13); 109.0411 (1) | 1) 1.11E-06  2) 5.91E-13  3) - |  | https://doi.org/10.1002/mnfr.201200777  analytical standard |
|  |  |  |  | 625.1179 (100)  *266.1213 (15)*  *267.1245 (1.5)* | [M+H]^+^ | POS |  |  |  |  |
| M27 | Indoxyl sulfate | 4.19 | C_8_H_7_NO_4_S  213.0096  Level I | 212.0023 (100)  *213.0053 (11)*  *213.9973 (9)*  **582.0910 (15)**  ***583.0943(8)*** | [M-H]^-^ | NEG | **MS/MS^2^ 582**: 212.0053 (100);  **MS/MS^2^ 582- 212**: 132.0457 (100) | 1) 8.87E-02  2) 2.02E-03  3) |  | Analytical standard |
|  |  |  |  | n.a | n.a | POS | n.a |  |  |  |
| M28 | Pipecolic acid  betaine | 4.0 | C_8_H_15_NO_2_  157.11027  Level III | n.a | n.a | NEG | n.a | - | 1) -  2) 3.29E-04  3) | doi:10.3945/jn.114.196840 |
|  |  |  |  | **158.1172 (100)**  *159.1204 (10)*  **112.1117 (40)**  140.1066 (20) | [M+H]^+^  [M+H-H_2_O-CO]^+^  [M+H-H_2_O]^+^ | POS | - |  |  |  |
| **Fatty acids and their metabolites** | | | | | | | | | | |
| M29 | Hydroxy fatty acid glucuronide  (OH)C11:3 | 4.99 | C_17_H_24_O_9_  372.1420  Level III | n.a | n.a | NEG | n.a | 1)2.54E-02  2)7.86E-04  3)- | - |  |
|  |  |  |  | **373.1490 (100)**  ***374.1520 (21)***  197.1170 (15)  391.1790 (25)  *390.1755 (8)* | [M+H]^+^  [M+H-GLC]^+^  [M+NH_4_]^+^ | POS | **MS/MS^2^ 373**: 197.1170 (100) |  |  |  |
| M30 | Hydroxy dicarboxylic fatty acid glucuronide  (OH)C14:1-GLC | 5.27 | C_20_H_32_O_11_  C_12_H_18_O_5_+GLC  448.1945  Level III | **447.1867 (100)**  ***448.1900 (26)*** | [M-H]^-^ | NEG | **MS/MS^2^ 447:** 271.1548(100)[M-H-GLC]^-^; 175.0249(15)[GLC-H]-; | 1) 8.71E-03  2) 5.91E-13  3)- | - |  |
|  |  |  |  | n.a | n.a | POS | n.a |  |  |  |
| M31 | Dihydroxy dicarboxylic fatty acid  (OH)_2_-C14:0 | 6.08 | C_14_H_26_O_6_  290.1729  Level III | **289.1652 (100)**  ***290.1686 (16)*** | [M-H]^-^ | NEG | **MS/MS^2^ 289:** 271.1551 (100); 253.1445 (10); 227.1653 (40); 209.1547 (20); 171.1024 (5); 125.0973 (3); 120.0827 (1)  **MS/MS^3^ 289-271:** 227.1649 (100); 209.1541 (20) | 1) 7.38E-04  2) 6.62E-11  3) - | - |  |
|  |  |  |  | 291.1801 (100) | [M+H]^+^ | POS |  |  |  |  |
| M32 | Hydroxy fatty acid glucuronide  (OH)C12:2-GLC | 7.06 | C_18_H_28_O_9_  388.1733  Level III | **387.1655 (100)**  ***388.1689 (18)***  ***389.1711 (3)***  **325.1651 (18)**  **775.3377 (12)**  ***776.3410 (6)*** | [M-H]^-^  [2M-H]- | NEG | **MS/MS^2^ 387**: 211.1335 (100); 193.0356(80); 175.0250(15) | 1) 5.08E-02  2) 3.64E-04  3)- | - |  |
|  |  |  |  | **195.1377 (100)**  ***196.1410 (11)***  **297.1671 (12)**  **213.1483 (80)**  ***214.1517 (14)***  **406.2067 (50)**  ***407.2102 (10)***  **411.1620 (20)**  ***412.1655 (4)***  **389.1830 (33)**  ***390.1837 (7)***  **117.0543 (11)** | [M+H-GLC-H_2_O]^+^  [M+H-GLC]^+^  [M+NH_4_]^+^  [M+Na]^+^  [M+H]^+^ | POS | **MS/MS^2^ 389**: 195.1377(100); 213.1483(40)  **MS/MS^2^ 411**: 235.1303(100);  **MS/MS^3^ 389-213:** 195.1377(100); 135.1166(13);177.1270(6);  **MS/MS^4^ 389-213-195:** 177.1273(100); 135.1166(50); 111.0438(34); 167.1428(34); 159.1167(22); 149.1325(26); 83.0490(21); |  |  |  |
| M33 | Dicarboxylic fatty acid (OH)C20:0 | 8.43 | C_20_H_38_O_5_  358.2719  Level III | **357.2647 (100)**  ***358.2679 (24)*** | [M-H]^-^ | NEG | **MS/MS^2^ 357:** 313.2748 (100) | 1) 5.91E-13  2) 5.91E-13  3) - | - |  |
|  |  |  |  | - | [M+H]^+^ | POS | **-** |  |  |  |
| M34 | Dicarboxylic fatty acid (OH)C20:1 | 8.60 | C_20_H_36_O_5_  356.2563  Level III | **355.2489 (100)**  *356.2524 (24)* | [M-H]^-^ | NEG | **MS/MS^2^ 355:** 311.2588 (100); 213.1859 (30) | 1) 5.91E-13  2) 5.91E-13  3) - |  |  |
|  |  |  |  | - | [M+H]^+^ | POS | **-** |  |  |  |
| M35 | Dicarboxylic fatty acid  (OH)_2_-C18:0 | 7.99 | C_18_H_34_O_6_  346.2355  Level III | **345.2285 (100)**  *346.2317 (21)* | [M-H]^-^ | NEG | **MS/MS^2^ 345:** 327.2172 (100) [M-H-H_2_O]^-^; 309.2070 (20) [M-H-2xH_2_O]^-^; 291.1953 (10) [M-H-C_4_H_6_]^-^; 265.2170 (20) [M-H-CO_2_-2xH_2_O]^-^; 247.2062 (19) [M-H-CO_2_-3xH_2_O]^-^; | 1) 6.38E-08  2) 4.73E-12  3) - |  |  |
|  |  |  |  | 347.2427 (100)  *348.2460 (21)* | [M+H]^+^ | POS | n.a |  |  |  |
| **Unknowns** | | | | | | | | | | |
| M36 | C_9_H_14_O_5_+GLC  Unknown metabolite | 2.00 | C_15_H_22_O_11_  378.1162  Level IV | **377.1089 (100)**  ***378.1121 (15)*** | [M-H]^-^ | NEG | **MS/MS^2^ 377:** 113.0248 (70); 175.0257 (100) | 1) 1.11E-06  2) 1.88E-04  3) - | - |  |
|  |  |  |  | 379.1233 (100)  *380.1267 (15)* | [M+H]^+^ | POS | **MS/MS^2^ 379:** 203.0910 (50)[M+H-GLC]+; 361.1129 (100); 341.0279 (25) |  |  |  |
| M37 | C_11_H_16_O_5_+SO_3_  Unknown metabolite | 2.39 | C_11_H_16_O_8_S  308.0566  Level IV | **307.0490 (100)**  ***308.0528 (14)***  *309.0455 (7)* | [M-H]^-^ | NEG | **MS/MS^2^ 307:** 227.0923 (100); 120.0761 (8) | 1) 6.38E-08  2) 4.71E-10  3) - | - |  |
|  |  |  |  | n.a | [M+H]^+^ | POS | n.a |  |  |  |
| M38 | C_10_H_14_O_4_ +SO_3_  Unknown metabolite | 2.48 | C_10_H_14_O_7_S  278.0460  Level IV | **277.0388 (100)**  **278.0422 (10)** | [M-H]^-^ | NEG | **MS/MS^2^ 277:** 197.0820 (100) [M-H-SO3]- | 1) 1.44E-03  2) 3.52E-05  3) - | - |  |
|  |  |  |  | n.a | - | POS |  |  |  |  |
| M40 | C_19_H_20_O_10_  Unknown metabolite | 5.90 | C_19_H_20_O_10_  408.1056  Level IV |  |  | NEG |  | 1) 1.42 E-12  2) 2.53 E-8  3) |  |  |
|  |  |  |  | **426.1388 (100)**  *427.1433 (16)*  233.0806 (80) | [M+NH_4_]^+^  [M+H-NH_4_-GLC]^+^ | POS | **MS/MS^2^ 426:** 233.0806 (100) [M+H-NH_4_-GLC]^+^ |  |  |  |
| M41 | Unknown metabolite | 7.71 | Level IV | **674.3541 (100)**  *675.3575 (42)*  *676.3631 (6)* | [M-H]^-^ | NEG | **MS/MS^2^ 674:** 498.3217 (100) [M-H-GLC]^-^ |  | 1) 4.28E-5  2) 7.82E-4  3) |  |
|  |  |  |  | **674.3540 (100)**  *675.3575 (42)*  *376.3631 (6)* | [M+H]^+^ | POS |  |  |  |  |
| M42 | Unknown metabolite | 8.67 | Level IV | **656.3439 (100)**  *657.3476 (40)*  *658.3466 (10)* | [M-H]^-^ | NEG | **MS/MS^2^ 656:** 480.3135 (100) [M-H-GLC]^-^ |  | 1) 5.54E-4  2) 3.20E-4  3)- |  |
|  |  |  |  | **658.3567 (100)**  *659.3600 (40)*  *660.3751 (10)* | [M+H]^+^ | POS |  |  |  |  |
| M43 | Unknown metabolite | 9.25 | Level IV | **658.3594 8100)**  *659.3629 (40)*  *660.3658 (10)* | [M-H]^-^ | NEG | **MS/MS^2^ 656:** 482.3281 (100) [M-H-GLC]^-^ |  | 1)  2) 7.67 E-5  3) |  |
|  |  |  |  | **660.3725 (100)**  *661.3765 (40)*  *662.3791 (10)* | [M+H]^+^ | POS |  |  |  |  |
| M44 | Unknown metabolite | 7.40 | C_18_H_26_O_4_  306.1831  Level IV | **307.1899 (100)**  *308.1943 (20)* | [M-H]^-^ | NEG |  |  | 1)  2) 8.23E-3  3) |  |
|  |  |  |  | na | [M+H]^+^ | POS | na |  |  |  |

m/z marked in bold were statistically significant;

m/z italics are isotopes

GLC – Glucuronide loss C_6_H_8_O_6_; MW 176.0321

SULF – sulfate loss SO_3_

n.a – not available
