# Supplementary material for: Impact of wheat aleurone on biomarkers of cardiovascular disease, gut microbiota and metabolites in adults with high body mass index: a double-blind, placebo-controlled, randomized clinical trial
Source: Eur J Nutr. 2022 Mar 5;61(5):2651–71. doi: 10.1007/s00394-022-02836-9 (PMC9279244; doi:10.1007/s00394-022-02836-9)
Supplement: Supplementary file 2 — Supplementary file2 (DOC 56 KB) [file 394_2022_2836_MOESM2_ESM.doc]

**Supplementary Information Table 2** Individual bile acids concentration (microMolar) in fasting plasma before (V1) and after (V2) dietary supplementation as measured by GC-MS.

| **Treatment** | **Time** |  | **CA** | **CDCA** | **DCA** | **UDCA** | **LCA** | **GCA** | **GDCA** | **GCDCA** | **GLCA** | **GUDCA** | **TCA** | **TDCA** | **TCDCA** | **TLCA** | **TUDCA** |
| --- | --- | --- | --- | --- | --- | --- | --- | --- | --- | --- | --- | --- | --- | --- | --- | --- | --- |
| **AL** | **V1** | **Mean** | 0.16 | 0.83 | 0.22 | 0.03 | 0.008 | 0.06 | 0.17 | 0.32 | 0.005 | 0.03 | 0.01 | 0.02 | 0.03 | 0.05 | 0.0006 |
|  |  | **SD** | 0.22 | 1.10 | 0.23 | 0.02 | 0.004 | 0.05 | 0.19 | 0.33 | 0.005 | 0.03 | 0.02 | 0.03 | 0.04 | 0.11 | 0.0006 |
| **AL** | **V2** | **Mean** | 0.13 | 1.07 | 0.21 | 0.03 | 0.012 | 0.07 | 0.16 | 0.32 | 0.007 | 0.04 | 0.01 | 0.02 | 0.04 | 0.02 | 0.0007 |
|  |  | **SD** | 0.18 | 1.38 | 0.20 | 0.02 | 0.003 | 0.04 | 0.24 | 0.28 | 0.010 | 0.04 | 0.01 | 0.03 | 0.03 | 0.06 | 0.0002 |
| **PL** | **V1** | **Mean** | 0.12 | 1.20 | 0.23 | 0.03 | 0.007 | 0.05 | 0.19 | 0.25 | 0.007 | 0.03 | 0.01 | 0.02 | 0.03 | 0.03 | 0.0004 |
|  |  | **SD** | 0.18 | 1.38 | 0.20 | 0.02 | 0.003 | 0.04 | 0.24 | 0.28 | 0.010 | 0.04 | 0.01 | 0.03 | 0.03 | 0.06 | 0.0002 |
| **PL** | **V2** | **Mean** | 0.18 | 0.90 | 0.19 | 0.03 | 0.006 | 0.07 | 0.16 | 0.22 | 0.005 | 0.03 | 0.02 | 0.03 | 0.04 | 0.04 | 0.0007 |
|  |  | **SD** | 0.29 | 1.07 | 0.13 | 0.03 | 0.004 | 0.12 | 0.22 | 0.18 | 0.005 | 0.02 | 0.07 | 0.06 | 0.06 | 0.12 | 0.0005 |

AL=aleurone; PL=placebo. CA=cholic acid; CDCA=chenodeoxicholic acid; DCA= deoxicholic acid; UDCA= ursodeoxicholic acid; LCA= lithocholic acid; GCA=glycocholic acid; GDCA= glycodeoxicholic acid; GCDCA= glycochenodeoxicholic acid; GLCA= Glycolithocholic acid; GUDCA= glycoursodeoxicholic acid; TCA= taurocholic acid; TCDCA= taurochenodeoxicholic acid; TLCA= taurolithocholic acid; TUDCA= tauroursodeoxicholic acid.
