# Supplementary figures and images for: Impact of wheat aleurone on biomarkers of cardiovascular disease, gut microbiota and metabolites in adults with high body mass index: a double-blind, placebo-controlled, randomized clinical trial
Source: Eur J Nutr. 2022 Mar 5;61(5):2651–71. doi: 10.1007/s00394-022-02836-9 (PMC9279244; doi:10.1007/s00394-022-02836-9)

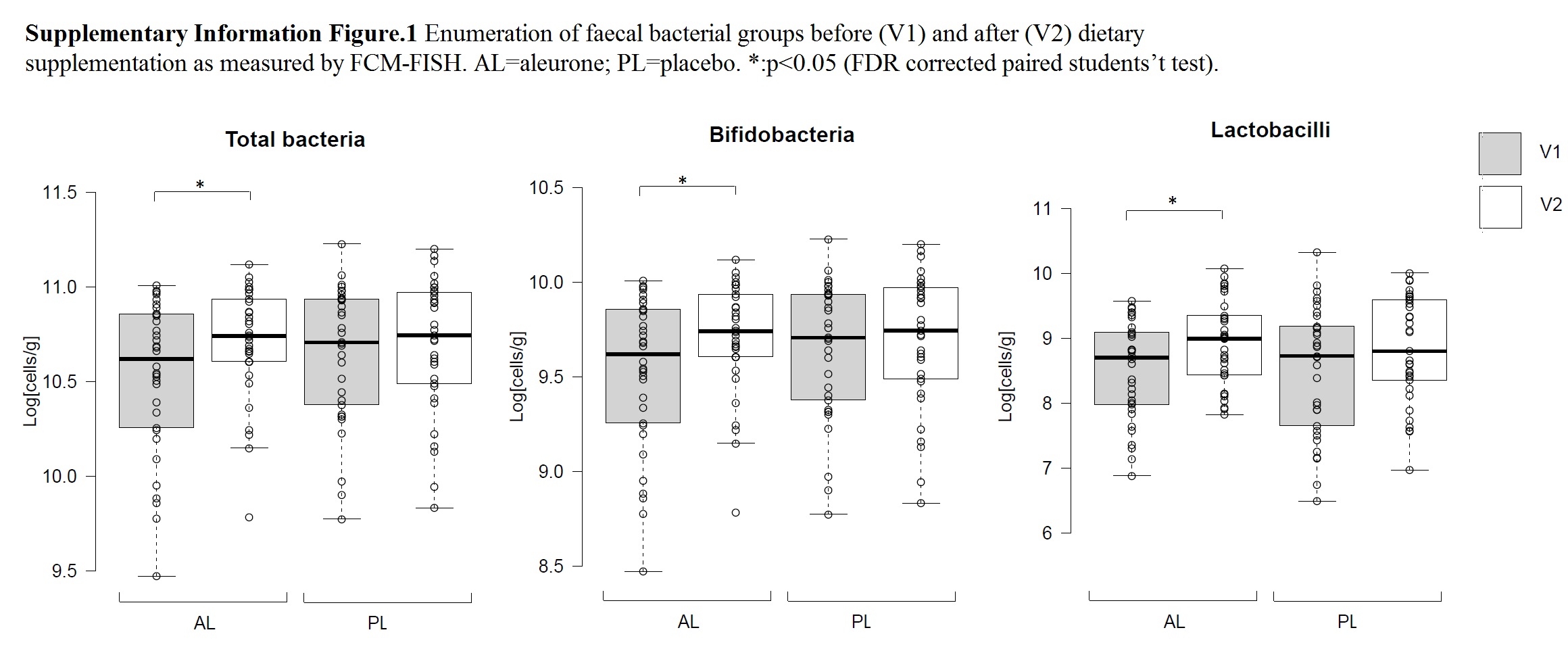

Supplement: Supplementary file 3 — Supplementary Information Figure.1 Enumeration of faecal bacterial groups before (V1) and after (V2) dietary supplementation as measured by FCM-FISH. AL=aleurone; PL=placebo. *:p<0.05 (FDR corrected paired students’ t test) [file 394_2022_2836_MOESM3_ESM.jpg]
